# Altered Brain Structure in Chronic Visceral Pain: Specific Differences in Gray Matter Volume and Associations With Visceral Symptoms and Chronic Stress

**DOI:** 10.3389/fneur.2021.733035

**Published:** 2021-10-20

**Authors:** Hanna Öhlmann, Laura Ricarda Koenen, Franziska Labrenz, Harald Engler, Nina Theysohn, Jost Langhorst, Sigrid Elsenbruch

**Affiliations:** ^1^Department of Medical Psychology and Medical Sociology, Ruhr University Bochum, Bochum, Germany; ^2^Institute of Medical Psychology and Behavioral Immunobiology, Center for Translational Neuro-and Behavioral Sciences, University Hospital Essen, University of Duisburg-Essen, Essen, Germany; ^3^Institute of Diagnostic and Interventional Radiology and Neuroradiology, University Hospital Essen, University of Duisburg-Essen, Essen, Germany; ^4^Department for Internal and Integrative Medicine, Sozialstiftung Bamberg, Bamberg, Germany; ^5^Department for Integrative Medicine, Medical Faculty, University of Duisburg-Essen, Essen, Germany; ^6^Department of Neurology, Center for Translational Neuro- and Behavioral Sciences, University Hospital Essen, University of Duisburg-Essen, Essen, Germany

**Keywords:** chronic visceral pain, gut-brain axis, inflammatory bowel disease, ulcerative colitis, irritable bowel syndrome, gray matter volume, voxel-based morphometry, chronic stress

## Abstract

Structural brain alterations in chronic pain conditions remain incompletely understood, especially in chronic visceral pain. Patients with chronic-inflammatory or functional bowel disorders experience recurring abdominal pain in concert with other gastrointestinal symptoms, such as altered bowel habits, which are often exacerbated by stress. Despite growing interest in the gut-brain axis and its underlying neural mechanisms in health and disease, abnormal brain morphology and possible associations with visceral symptom severity and chronic stress remain unclear. We accomplished parallelized whole-brain voxel-based morphometry analyses in two patient cohorts with chronic visceral pain, i.e., ulcerative colitis in remission and irritable bowel syndrome, and healthy individuals. In addition to analyzing changes in gray matter volume (GMV) in each patient cohort vs. age-matched healthy controls using analysis of covariance (ANCOVA), multiple regression analyses were conducted to assess correlations between GMV and symptom severity and chronic stress, respectively. ANCOVA revealed reduced GMV in frontal cortex and anterior insula in ulcerative colitis compared to healthy controls, suggesting alterations in the central autonomic and salience networks, which could however not be confirmed in supplemental analyses which rigorously accounted for group differences in the distribution of sex. In irritable bowel syndrome, more widespread differences from healthy controls were observed, comprising both decreased and increased GMV within the sensorimotor, central executive and default mode networks. Associations between visceral symptoms and GMV within frontal regions were altered in both patient groups, supporting a role of the central executive network across visceral pain conditions. Correlations with chronic stress, on the other hand, were only found for irritable bowel syndrome, encompassing numerous brain regions and networks. Together, these findings complement and expand existing brain imaging evidence in chronic visceral pain, supporting partly distinct alterations in brain morphology in patients with chronic-inflammatory and functional bowel disorders despite considerable overlap in symptoms and comorbidities. First evidence pointing to correlations with chronic stress in irritable bowel syndrome inspires future translational studies to elucidate the mechanisms underlying the interconnections of stress, visceral pain and neural mechanisms of the gut-brain axis.

## Introduction

Despite substantial individual and societal burden, chronic pain is often overlooked and remains incompletely understood, especially with respect to brain mechanisms relevant to pathophysiology, disease course, and treatment. Clinical conditions characterized by chronic visceral or pelvic pain are particularly understudied using brain imaging techniques. Dedicated visceral pain research is warranted not only given the unique clinical presentation of chronic visceral pain. Many afflicted patients do not experience pain arising from the viscera (i.e., inner organs such as the thorax, pelvis or abdomen) in isolation, but rather suffer from recurring episodes of abdominal pain or discomfort in concert with other gastrointestinal (GI) symptoms, such as bowel disturbances. Work in visceral pain is also called for in light of increasing knowledge about the specificity of visceral pain both in terms of psychological as well as central mechanisms. In contrast to somatic pain, visceral pain is perceived as more diffuse and more unpleasant, provokes more pain-related fear, may be more sensitive to stress (1–4), and, importantly, engages partly distinct functional brain responses, at least during acute experimental pain (2, 5). Finally, the clinical relevance of chronic visceral pain is enormous, with a prevalence that likely surpasses even that of chronic low back and neck-shoulder pain. Indeed, intermittent abdominal pain is experienced by 25 % of adults in the general population (6), and also constitutes the most prevalent GI symptom that causes outpatient clinic visits in the United States (7).

Numerous GI conditions are characterized by visceral pain and pain-related symptoms arising from the GI tract, together contributing to substantial psychological distress, functional disability, and healthcare costs (8). The most prominent GI conditions are traditionally classified as either structural diseases with a clear organic pathology, such as chronic-inflammatory bowel diseases (IBD), or as functional disorders lacking a clearly identifiable organic cause, like irritable bowel syndrome (IBS). IBD is a relapsing-remitting disease mainly characterized by chronic intestinal inflammation, with the localization of intestinal inflammation defining the specific diagnosis of ulcerative colitis (UC; primarily affecting the colon) and Crohn's disease (affecting various GI sites) (9). Of note, about 35% of patients with IBD experience abdominal pain and changes in bowel habits not only during active but also in phases of inactive disease, when the clinical presentation can mirror that of IBS (10). IBS is considered a bio-psycho-social disorder of gut-brain interaction, with unclear etiology yet long-standing appreciation for a crucial role of brain mechanisms relevant to visceral hypersensitivity and hypervigilance, interacting with peripheral factors like increased gut permeability and low-grade inflammation (11). Despite differences in etiology and pathophysiology, psychological factors, especially stress, play a major role in both IBD and IBS, in line with evolving concepts of the gut-brain axis (12, 13).

The role of psychological factors in acute and chronic visceral pain has inspired translational research elucidating the complex signaling pathways between the GI system and the brain, both in health and disease. There exist multiple connections between the gut and the central nervous system involving microbial, immunological, metabolic, hormonal, and neural processes (14). Chronic abdominal pain can be conceptualized as a dysregulation in this complex interplay (15). As the brain is a highly dynamic system, this dysregulation conceivably implies not only changes in functional but also structural brain imaging measures, in line with broad evidence of morphological brain alterations in various somatic chronic pain conditions (16–18). A meta-analysis by Cauda and colleagues revealed that different chronic pain conditions share alterations of gray matter volume (GMV) in regions of the default-mode, thalamus-basal ganglia and attention networks, while GMV changes in sensory networks are more variable and depend on the specific chronic pain condition (19). In chronic visceral pain, the presence and putative role of morphological brain changes has been much more extensively studied in IBS than in IBD. For IBS, systematic reviews support structural alterations in regions of the prefrontal, salience, emotional arousal and sensorimotor networks, with GMV decreases in the insular cortex and GMV increases in sensorimotor cortices as most consistent findings (20–22). In IBD, knowledge about altered brain morphology is very limited, especially in UC. Results are inconsistent, and mostly available for cohorts comprising only patients with Crohn's disease (23–27) or mixed samples of Crohn's disease and UC (28, 29). Only a single study focused exclusively on patients with UC (30), despite indications for differences in brain morphology and function between UC and Crohn's disease (29).

Furthermore, efforts to elucidate correlations between structural brain abnormalities and relevant pain-related GI symptoms, as well as with chronic stress as a major psychological factor relevant to the pathophysiology, disease course, and treatment in both IBS and IBD (31, 32), have rarely been accomplished. In IBS, structural alterations have been shown to correlate not only with GI symptoms, but also with psychological variables, including psychiatric comorbidities (33, 34), pain catastrophizing (34), and early trauma (35), but perceived chronic stress as a major factor has not been studied. This research gap also exists for IBD, with only a single existing study testing correlations between brain function (rather than structure) and acute stress (36). Given long-standing knowledge that chronic rather than acute stress is relevant to symptom exacerbation (37, 38) as well as to pain and health-related quality of life in IBD (39), attention to chronic stress in brain imaging studies is urgently called for.

To close research gaps in structural neuroimaging studies focused on patients with chronic visceral pain, we herein present results of parallelized voxel-based morphometry analyses accomplished in patients with UC and patients with IBS, compared to matched healthy control groups. In order to minimize effects of acute inflammation and severe symptoms characterizing phases of active disease, we only included patients in full remission or with very mild and stable disease activity, at the same time minimizing possible effects of medical treatments routinely necessary in these patients, especially during exacerbations. As a first step in the analysis strategy, voxel-based morphometry was accomplished to determine changes in GMV in each patient group compared to healthy controls, using whole-brain analyses given variability of findings and the widespread structural alterations observed in previous studies. Although alterations in GMV compared to controls were expected in both disorders, differences were hypothesized to be more pronounced and widespread in IBS than in UC given differences in the etiology and pathophysiology, especially regarding the presumably more prominent role of central mechanisms along the gut-brain axis in IBS. As a second step, we performed analyses aiming to address associations with symptom severity and chronic stress in each patient cohort compared to controls using multiple regression analyses. Given overlap in symptoms experienced by patients with UC and IBS and evidence for the role of stress in both disorders (12), it was hypothesized that both symptom severity and chronic stress are differentially related to GMV in both patient groups compared to healthy controls. Here, we expected effects in neural networks previously shown to be relevant to symptom intensity and psychological modulation of acute and chronic visceral pain [e.g., sensorimotor and emotional arousal networks; (20)].

## Methods

### Overview and Procedures

For the purposes of the present analyses, we used data from a total of *N* = 96 adult volunteers (*N* = 31 UC, *N* = 23 IBS, *N* = 44 healthy controls), acquired within two comprehensive visceral pain studies conducted by our group between the years 2015 and 2020 at the University Hospital Essen, Germany. Primary studies involved different emotional learning/memory tasks (data will be presented elsewhere), all accomplished subsequent to the acquisition of data analyzed herein. Importantly, all participants underwent sociodemographic, psychological and clinical characterization and structural magnetic resonance imaging (MRI) prior to other experimental manipulations. Highly standardized and parallelized procedures were implemented for recruitment, screening and all other assessments that are part of this report, all accomplished within the same biomedical research setting using the same MR scanner. Work was conducted in accordance with The Declaration of Helsinki, and studies were approved by the local Ethics Committee of the University Hospital Essen (protocol numbers. 10-4493; 16-7237). All volunteers gave written informed consent and received monetary compensation for participation.

### Inclusion and Exclusion Criteria

The screening process consisted of a standardized telephone screening, followed by an on-site visit with study staff, and completion of questionnaires (for details on questionnaires, see below). General exclusion criteria for all participants included age <18 or >65 years, body mass index <18 or >30, MRI-specific criteria like claustrophobia, pregnancy or ferromagnetic implants, and any evidence of structural brain abnormalities, verified by a neuroradiologist (author NT). Pregnancy was ruled out using a commercially available pregnancy test on the day of the MRI (Biorepair GmbH, Sinsheim, Germany, sensitivity 10 mIU/ml). For healthy controls, additional exclusion criteria included any known somatic or mental health condition, clinically-relevant anxiety or depression symptoms based on Hospital Anxiety and Depression Scale (HADS), or regular use of medications (except hormonal contraceptives, hormone replacement therapy, thyroid medication, irregular over-the-counter non-prescription drugs). For the UC group, only patients in clinical remission or with very low ongoing disease activity were included to avoid interference of active disease with study-related procedures, and to minimize putative effects of acute inflammation (or medical treatments required during phases of disease exacerbation) on study-related measures of interest acquired herein. Clinical disease activity was assessed based on symptom reports, initially evaluated in a structured screening interview, and then quantified with the Clinical Colitis Activity Index [CAI; (40)]. In addition, levels of fecal calprotectin were quantified, providing a non-invasive marker of intestinal inflammation (41), with an established reliable cut-off value indicating clinical remission below 150 μg (42). Treatment with systemic glucocorticoids within 4 weeks of the study were exclusionary. Other concomitant medications, which were continued as prescribed by the treating physician, were recorded. For IBS, symptom-based confirmation of diagnostic criteria was based on ROME IV criteria (43). Regular prescribed or non-prescribed IBS-related medications including low-dose treatment with antidepressants were not discontinued for the study. While minor and stable (or successfully treated) psychological symptoms, such as mild anxiety or depression symptoms (including elevated HADS scores) were not exclusionary, patients with diagnosed, more severe psychiatric comorbidities were excluded. Note that given frequent reporting of additional extraintestinal pain symptoms in IBS and IBD (44, 45), patients who reported such symptoms in addition to symptoms of their primary GI diagnoses were not excluded, but other types of chronic or recurring pain symptoms and chronic pain diagnoses were recorded. For all patients, an existing and confirmed diagnosis (of the respective GI disorder) established at least 1 year prior to recruitment for this study was required.

### Clinical Symptom Questionnaires

In all participants, GI symptoms were quantified with a standardized questionnaire that we routinely use in our group as it is applicable across visceral pain conditions as well as in healthy volunteers [who also experience such symptoms, albeit less frequently or intensely; (46)]. A range of typical GI symptoms (i.e., diarrhea, constipation, vomiting, nausea, lower abdominal pain, upper abdominal pain, heartburn, post-prandial fullness, bloating, loss of appetite) in the previous 3 months is assessed using a Likert-type response scale (0 = experience never, 1 = experience once or twice per month, 2 = experience once or twice per week, 3 = experience more than twice a week). The total sum score was calculated for analyses. Given the specific interest in visceral pain herein, individual responses on the items for upper and lower abdominal pain, respectively, are additionally provided for a more specific characterization of GI symptoms in each group (Table 1). For patients with IBS, current bowel alteration(s) and bowel symptom subtyping (i.e., diarrhea-predominant, constipation-predominant, mixed) were also accomplished based on the GI symptom questionnaire. Patients with UC additionally completed the CAI (40) to assess clinical disease activity. The CAI consists of 6 items capturing a range of typical UC symptoms (i.e., increase in stool frequency, bloody stools, abdominal pain, temperature due to colitis, extraintestinal manifestations, and the investigator's global assessment of symptomatic state) as well as 1 item concerning laboratory results (i.e., erythrocyte sedimentation rate and hemoglobin). Hemoglobin is relevant, as anemia is the most common complication in IBD associated with disease activity, and the erythrocyte sedimentation rate is a biomarker of inflammation. Based on the total sum score, disease activity can be classified into inactive (i.e., remission; ≤ 4), mild activity (5–10), moderate activity (11–17) and high activity (≥18) with a maximum score of 26 (47). However, laboratory assessments were not available for the entire sample of UC patients (missing for *N* = 13 patients), which is why we provide CAI average scores computed based on 6 items for all patients for consistency. In results, we refer to this measure as symptom-based CAI for clarity.

**Table 1:** Sociodemographic, clinical, and psychological self-report data.

	**UC (*N* = 31)**	**HC_UC_ (*N* = 31)**	***p*** ^*^	**IBS (*N* = 23)**	**HC_IBS_ (*N* = 23)**	***p*** ^*^
Sex (Females, *N*)	26 F	31 F	–	23 F	23 F	–
Age, years	41.45 ± 12.82	41.61 ± 11.91	0.959	46.91 ± 10.92	43.74 ± 12.17	0.357
BMI	23.61 ± 3.80	22.33 ± 2.53	0.124	23.13 ± 4.02	23.31 ± 2.72	0.861
Gastrointestinal symptoms (total sum)	8.45 ± 6.47	3.35 ± 2.68	<0.001	15.09 ± 4.88	3.70 ± 3.15	<0.001
Lower abdominal pain (1 item)	1.06 ± 1.06	0.32 ± 0.54	0.001	1.87 ± 1.06	0.43 ± 0.66	0.001
Upper abdominal pain (1 item)	0.48 ± 0.89	0.16 ± 0.37	0.07	0.78 ± 0.85	0.13 ± 0.34	0.002
Psychological distress (HADS total)	9.65 ± 4.72	6.42 ± 3.82	0.004	15.09 ± 6.09	6.48 ± 4.38	<0.001
Anxiety symptoms (HADS_A)	6.32 ± 2.82	3.77 ± 2.32	<0.001	9.09 ± 3.27	3.61 ± 2.54	<0.001
Depression symptoms (HADS_D)	3.32 ± 2.83	2.65 ± 2.17	0.295	6.00 ± 3.30	2.87 ± 2.47	0.001
Chronic stress (TICS)	19.32 ± 8.98	16.32 ± 9.22	0.199	25.17 ± 8.58	15.04 ± 9.44	<0.001

* *Results of two-tailed independent-samples t-tests comparing each patient group and the matched control group. Data are shown as mean ± standard deviation, unless otherwise specified. UC, ulcerative colitis; HC_UC_, matched healthy control group for UC group; IBS, irritable bowel syndrome; HC_IBS_, matched healthy control group for IBS group; BMI, body mass index; HADS, hospital anxiety and depression scale; HADS_A, HADS anxiety subscale; HADS_D, HADS depression subscale; TICS, trier inventory of chronic stress*.

### Chronic Stress and Psychological Distress

Chronic stress was assessed by the 12-item screening scale of the Trier Inventory of Chronic Stress [TICS-SSCS; (48)]. The scale evaluates individual experiences with chronic stressors in everyday life and provides a reliable global measure of perceived stress during the last 3 months (49). Likert-scale response options are “never” (0), “rarely” (1), “sometimes” (2), “often” (3), and “very often” (4), with a total score ranging from 0 to 48, and higher scores indicating greater perceived presence and frequency of chronic stressors. Note that we chose this questionnaire specifically for its applicability not only to research in clinical populations but also in healthy volunteers, expanding on our early work on the role of chronic stress in the context of visceral pain (50).

In addition, the Hospital Anxiety and Depression Scale [HADS; (51)] was used as screening tool, and to provide a clinically-relevant and widely-used measure suitable for a characterization of patient groups with respect to psychological distress. The HADS consists of two subscales with 7 items measuring anxiety (HADS_A) and depression (HADS_D), respectively. For each subscale, available cut-off values differentiate between non-cases (subscale score <8), potential cases (subscale score 8–10), and probable cases (subscale score ≥11) of anxiety and depression (52). For the purposes of sample characterization, in addition to the two subscale scores, we provide mean total scores (HADS Total), which can range from 0 to 42 with higher scores indicating higher levels of overall psychological distress.

All questionnaire data and other self-report variables were analyzed using IBM SPSS Statistics 27 (IBM Corporation, Armonk, NY). Group comparisons were accomplished using independent-samples *t*-tests, and data are reported as mean ± standard deviation, unless indicated otherwise.

### Magnetic Resonance Imaging and Voxel-Based Morphometry

Structural images were acquired on a 3 Tesla MR scanner using a 32-channel head coil (Skyra, Siemens Healthcare, Erlangen, Germany). All data were acquired on the identical scanner, and used one of two 3D-MPRage T1-weighted sequences with very similar yet not identical acquisition parameters: sequence 1 [repetition time (TR) 1,900 ms, echo time (TE) 2.13 ms, flip angle 9°, field of view (FOV) 239 × 239 mm^2^, voxel size 0.9 × 0.9 × 0.9 mm^3^]; sequence 2 [TR 1,770 ms, TE 3.24 ms, flip angle 8°, FOV 256 × 256 mm^2^, voxel size 1 × 1 × 1 mm^3^]. All group analyses were accomplished after a matching of healthy controls (based on the entire sample of *N* = 44) to each individual patient group, providing dedicated control groups for UC and IBS, respectively, referred to subsequently as HC_UC_ and HC_IBS_. The matching procedure was based on MR scanning sequence and age, accounting for the slightly different acquisition parameters of the two sequences. Note that the data included for analyses of IBS vs. HC_IBS_ were all acquired with sequence 1; analyses of UC vs. HC_UC_ had equal number of patients and healthy controls measured with sequence 1 (*N* = 13 UC, *N* = 13 HC_UC_) and 2 (*N* = 18 UC, *N* = 18 HC_UC_). The acquired images were pre-processed and analyzed with the CAT12 toolbox (Structural Brain Mapping group, Jena University Hospital, Jena, Germany) and SPM12 (Statistical Parametric Mapping, Wellcome Center for Human Neuroimaging, UCL Queen Square Institute of Neurology, London, UK) implemented in Matlab R2020a (MathWorks Inc., Natick, MA, USA). The analysis followed the standard protocol for this toolbox (http://www.neuro.uni-jena.de/cat12/CAT12-Manual.pdf) using default settings and parameters, unless otherwise specified. The main processing steps included the segmentation of voxels into gray matter (GM), white matter (WM) and cerebrospinal fluid (CSF), and normalization using optimized shooting registration. After pre-processing, the homogeneity of the sample was checked by inspecting the correlation between all volumes to ensure data quality. As all images showed high correlation values (>0.86), no images were excluded from further analysis. Modulated normalized GM maps were smoothed with a Gaussian kernel of 8 mm (FWHM). The smoothed images were used for further analysis to test for regional GMV differences between groups. Atlas labeling was based on MRI scans originating from the Open Access Series of Imaging Studies (OASIS) project. The labeled data were provided by Neuromorphometrics under academic subscription (Neuromorphometrics, Inc., Somerville, MA, USA). The total intracranial volume was determined for each subject, as it is an important confounding variable in voxel-based morphometry.

All whole-brain statistical analyses were performed within the CAT12 environment. To increase sensitivity and to avoid the arbitrary choice of an initial cluster-forming threshold, the Threshold Free Cluster Enhancement [TFCE; (53, 54)] toolbox was used for all analyses (Structural Brain Mapping group, Jena University Hospital, Jena, Germany). In a first step, two analyses of covariance (ANCOVA) were run to compare the GMV between each patient group and matched healthy controls with total intracranial volume and age as covariates of no interest. Note that T1-sequence was additionally included as covariate of no interest for the comparison of UC vs. HC_UC_. For all ANCOVAs, we report significant results corrected for multiple comparisons [using family-wise error (FWE) correction of alpha, set at *p* < 0.05].

In a second step, four multiple regressions were calculated (i.e., two for each patient group vs. controls) to test for group interactions in the correlation of GMV with GI symptoms and chronic stress, respectively, controlling for total intracranial volume, age (and sequence where appropriate) as covariates of no interest. For these analyses, we report FWE-corrected results as well as results without correction applying an alpha level of *p* < 0.001. For each cluster identified by multiple regression analysis, the estimated GMV of its peak brain region was extracted and transferred to SPSS. As exploratory analysis, we examined correlations of GMV within brain regions identified by multiple regression analyses and GI symptoms and chronic stress, respectively, within each group using partial correlation analyses. To this end, the extracted tissue volumes within anatomical regions and GI symptoms and chronic stress were regressed based on total intracranial volume, age, and sequence (where appropriate). Correlational analyses were then accomplished and plotted using RStudio (version 1.2.5001, RStudio PBC).

Supplemental analyses were carried out as follows (all results reported within Supplementary Material): Firstly, as sex was not equally distributed in patients with UC and HC_UC_, all analyses were re-computed in a subsample comprising only women, i.e., after exclusion of 5 male patients and their 5 age-matched female controls. Secondly, to indirectly address whether patterns of GMV alterations in patients with UC and IBS are disease-specific, further data and results are provided (details on approach provided in Supplementary Material). The approach included extracting and plotting GMV of the clusters identified in the comparison of one patient group and matched healthy controls in the other patient group and matched healthy controls, and using these clusters as regions of interest (ROI) in ROI-based analyses.

## Results

### Sociodemographic, Clinical, and Psychological Characteristics

As per matching of healthy controls to patient group based on age and T1-scanning sequence, the final samples we report upon consisted of *N* = 31 UC vs. *N* = 31 HC_UC_ and *N* = 23 IBS vs. *N* = 23 HC_IBS_ (with *N* = 2 healthy controls excluded during matching and an overlap of *N* = 12 healthy controls in both control groups). As intended by matching and consistent with stringent screening for abnormal BMI, no differences between the patient and control groups were evident in age or BMI (Table 1). In both patient groups, GI symptoms were expectedly significantly increased compared to healthy controls, as were reports of abdominal pain, especially in the lower abdominal region. For the UC patient group, inclusion of patients in remission or with only mild disease activity was successful, as confirmed by a symptom-based average CAI of 1.48 (*SD* = 1.99), and a median fecal calprotectin concentration of 41.88 μg (IQR = 83.74 μg). IBD-related medications continued as prescribed by the treating physician included aminosalicylates (*N* = 20), local corticosteroids (*N* = 2), TNF-α blocker (*N* = 2), and azathioprine (*N* = 2). Few patients reported additional extraintestinal pain symptoms (fibromyalgia, *N* = 2; migraine, *N* = 2; arthritis, *N* = 1). Patients with IBS reported different bowel habit disturbances, as is typical for this condition, with diarrhea-predominant (*N* = 9), constipation-predominant (*N* = 4), mixed IBS (*N* = 9), or unspecified (*N* = 1). IBS-related medications included selective serotonin reuptake inhibitors (*N* = 1), muscarine receptor antagonists (*N* = 2), and loop diuretics (*N* = 1). Extraintestinal pain symptoms were reported by some patients (fibromyalgia, *N* = 3; migraine, *N* = 1, arthritis, *N* = 2). Regarding psychological variables, significantly higher levels of psychological distress based on HADS total score were observed in both patient groups, while only patients with IBS reported significantly more chronic stress when compared to controls (Table 1).

### Group Differences in Brain Morphology

**Figure 1:**
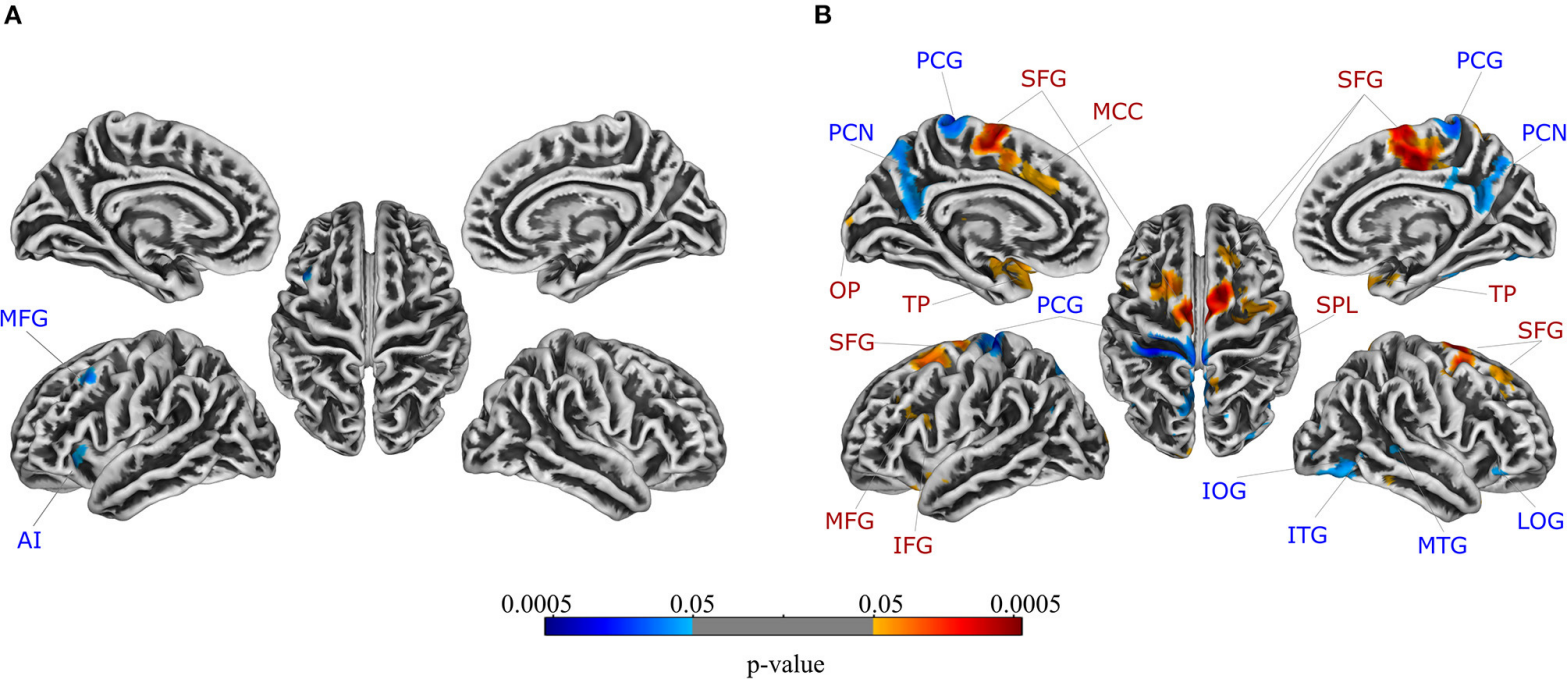
Regions in which gray matter volume was lower in patients compared to healthy controls are depicted in blue, while regions in which gray matter volume was higher in patients compared to healthy controls are shown in red for patients with **(A)** ulcerative colitis and **(B)** irritable bowel syndrome. FWE-correction was applied at the significance level of *p* < 0.05. Axial slices are provided in Supplementary Figures 1, 3, respectively. For details, see Table 2. AI, anterior insula; IFG, inferior frontal gyrus; IOG, inferior occipital gyrus; ITG, inferior temporal gyrus; LOG, lateral orbital gyrus; MCC, middle cingulate gyrus; MFG, middle frontal gyrus; MTG, middle temporal gyrus; OP, occipital pole; PCG, postcentral gyrus; PCN, precuneus; SFG, superior frontal gyrus; SPL, superior parietal lobule; TP, temporal pole.

For the comparison between patients with UC and healthy controls, the ANCOVA identified two clusters in which GMV was significantly lower in patients with UC (Table 2). These clusters comprised the left middle frontal gyrus and left anterior insula, respectively. In addition to a rendered view (Figure 1A), the two clusters are visualized on axial slices to enable a more precise localization (Supplementary Figure 1). Each cluster's extracted GMV was plotted for patients with UC and matched control groups to provide data on the single-subject level (Supplementary Figure 2). However, it should be kept in mind that these plots cannot visualize the correction for total intracranial volume, age, and sequence that was applied in the ANCOVA. In the reversed contrast, no clusters demonstrating higher GMV in patients with UC compared to healthy controls yielded significance.

**Table 2:** Results of whole-brain ANCOVAs comparing gray matter volume in the two patient groups to healthy controls.

**Brain region**	**H**	**k**	**TFCE**	***p*** ***_FWE_***	**x**	**y**	**z**
**UC < HC_UC_**							
Middle frontal gyrus	L	521	323.01	0.029	−42	20	45
Anterior insula	L	334	273.41	0.032	−23	23	−9
**IBS < HC_IBS_**							
Postcentral gyrus	L	2,936	553.22	0.002	−29	−30	72
Precuneus	L	2,703	243.29	0.02	2	−75	53
Inferior temporal gyrus	R	755	255.01	0.018	51	−41	−32
Middle temporal gyrus	R	144	155.41	0.046	48	−41	−3
Lateral orbital gyrus	R	201	170.81	0.04	47	29	−18
Inferior occipital gyrus	R	805	226.23	0.023	56	−69	−12
	R	264	180.43	0.036	44	−89	5
**IBS > HC_IBS_**							
Superior frontal gyrus	L	9,185	644.58	0.001	12	11	72
	R	631	208.35	0.028	24	45	47
	R	236	189.4	0.033	23	35	30
Middle frontal gyrus	L	109	176.01	0.038	−42	41	33
	L	110	170.45	0.04	−38	23	20
Inferior frontal gyrus (opercular part)	L	184	173.6	0.039	−59	14	21
Temporal pole	L	3,005	230	0.022	−24	3	−17
	R	383	196.58	0.031	33	26	−39
	R	254	178.35	0.037	35	−2	−30
Superior parietal lobule	R	163	205.23	0.028	9	−54	69
Middle cingulate gyrus	L	519	179.19	0.037	−11	24	26
Occipital pole	L	296	191.12	0.032	−14	−96	8

*Results are FWE-corrected at p < 0.05 and the total intracranial volume, age, and sequence (where appropriate) were included as covariates of no interest. H, hemisphere; L, left; R, right; k, cluster size; TFCE, threshold-free cluster enhancement; x, y, z, MNI coordinates; UC, ulcerative colitis; HC_UC_, matched healthy control group for UC group; IBS, irritable bowel syndrome; HC_IBS_, matched healthy control group for IBS group*.

For the comparison between patients with IBS and healthy controls, the ANCOVA identified seven clusters with significantly lower GMV in patients with IBS (Table 2). These clusters encompassed the left postcentral gyrus, left precuneus, right lateral orbital gyrus, right inferior temporal gyrus, right middle temporal gyrus, and right inferior occipital gyrus, respectively. Clusters are depicted in a rendered view (Figure 1B, blue color scale) as well as axial slices (Supplementary Figure 3). Again, each cluster's GMV was extracted and plotted for patients with IBS as well as matched controls (Supplementary Figure 4A). In contrast, GMV was significantly higher in patients with IBS compared to healthy controls in 12 clusters including the bilateral temporal pole, bilateral superior frontal gyrus, left middle cingulate gyrus, left middle frontal gyrus, left opercular part of the inferior frontal gyrus, left occipital pole, and right superior parietal lobule, respectively. These results are visualized using a rendered view (Figure 1B, red color scale) and axial slices (Supplementary Figure 3), and GMV of these clusters was extracted and plotted for IBS patients as well as matched controls (Supplementary Figure 4B).

### Associations Between Gray Matter Volume and Gastrointestinal Symptoms

**Figure 2:**
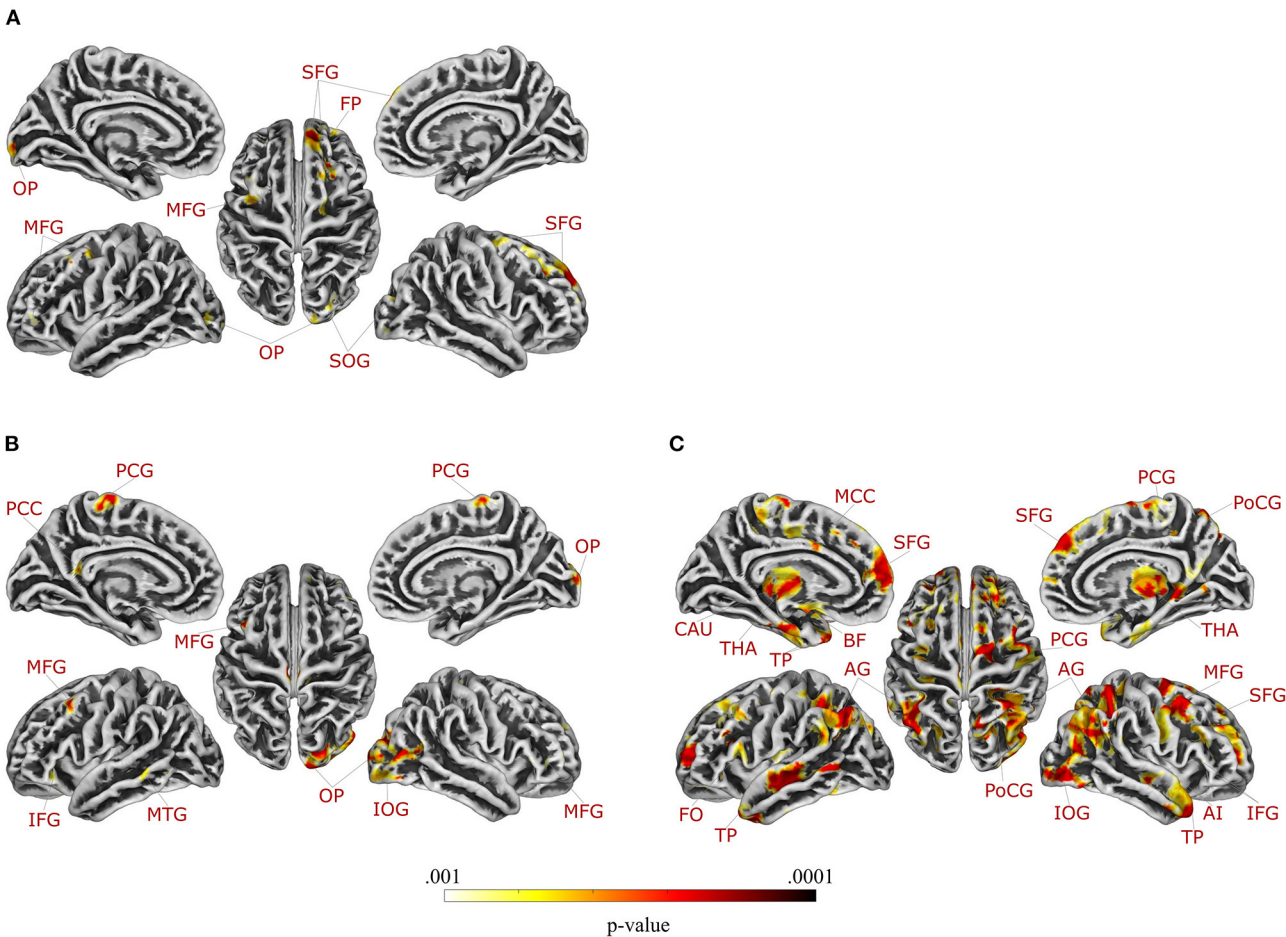
Regions in which gray matter volume was differentially correlated with gastrointestinal symptoms in patients with **(A)** ulcerative colitis and **(B)** irritable bowel syndrome compared to healthy controls, and **(C)** regions in which gray matter volume was differentially correlated with chronic stress in patients with irritable bowel syndrome compared to healthy controls (applying a significance level of *p* < 0.001, uncorrected for multiple comparisons). Axial slices are provided in Supplementary Figures 5–7. For details, see Tables 3, 4. AI, anterior insula; AG, angular gyrus; BF, basal forebrain; CAU, caudate nucleus; FO, frontal operculum; FP, frontal pole; IFG, inferior frontal gyrus; IOG, inferior occipital gyrus; MCC, middle cingulate gyrus; MFG, middle frontal gyrus; MTG, middle temporal gyrus; OP, occipital pole; PCC, posterior cingulate gyrus; PCG, precentral gyrus; PoCG, postcentral gyrus; SFG, superior frontal gyrus; SOG, superior occipital gyrus; THA, thalamus; TP, temporal pole.

Multiple regression analysis testing correlations of GMV and GI symptoms in patients with UC and HC_UC_ revealed a significant interaction effect between group and GI symptoms in two clusters located in the right superior frontal gyrus (Table 3). Supplemental partial correlational analyses between extracted GMV for this region and GI symptoms revealed a negative correlation in UC and a positive correlation in HC_UC_, suggesting that greater GI symptoms correlated with reduced GMV in superior frontal gyrus only in patients (details and partial correlation plots in Supplementary Figure 8). Note that multiple regression analysis performed without correction for multiple comparisons revealed an interaction effect between group and GI symptoms in nine clusters (at *p* < 0.001), comprising additional frontal and occipital regions (Figure 2A, Table 3). These results are additionally visualized on axial slices to enable a more precise localization (Supplementary Figure 5).

**Table 3:** Results of whole-brain multiple regression correlating gray matter volume and gastrointestinal symptoms in patients with UC and patients with IBS compared to healthy controls.

**Brain region**	**H**	**k**	**TFCE**	***p*** ^*^	**x**	**y**	**z**
**UC < HC_UC_**							
Superior frontal gyrus	R	462	1,359.98	0.037	12	56	24
	R	130	1,338.89	0.039	18	39	35
Middle frontal gyrus	L	726	1,073.7	<0.001	−35	2	59
	L	113	871.09	0.001	−47	53	−3
Frontal pole	R	126	866.6	0.001	32	65	−8
Superior occipital gyrus	R	178	1,051.01	0.001	30	−87	20
Occipital pole	L	462	1,013.21	<0.001	−18	−95	−5
	R	165	1,050.94	0.001	17	−96	9
**IBS < HC_IBS_**							
Occipital pole	R	130	1,538.2	0.038	20	−93	6
Middle frontal gyrus	L	328	1,135.04	0.001	−44	17	41
	R	109	1,112.4	0.001	38	62	2
Inferior frontal gyrus (orbital part)	L	350	1,490.26	0.001	−42	23	−5
Precentral gyrus	L	860	1,131.42	<0.001	9	−27	66
Middle temporal gyrus	L	151	829	0.001	−69	−42	9
Posterior cingulate gyrus	L	117	684.76	0.001	−8	−48	15
Inferior occipital gyrus	R	3,859	1,538.9	<0.001	20	−93	6

* *P-values from FWE-corrected analyses are underlined; all other values indicate results from uncorrected analyses (interaction group × GI symptoms). H, hemisphere; L, left; R, right; k, cluster size; TFCE, threshold-free cluster enhancement; x, y, z, MNI coordinates; UC, ulcerative colitis; HC_UC_, matched healthy control group for UC group; IBS, irritable bowel syndrome; HC_IBS_, matched healthy control group for IBS group*.

In patients with IBS and controls, multiple regression analysis testing correlations between GMV and GI symptoms resulted in a significant interaction effect between group and GI symptoms in one cluster in the right occipital pole (Table 3). Supplemental partial correlational analyses between extracted GMV for this region and GI symptoms revealed a negative correlation in IBS and a positive correlation in HC_IBS_, suggesting that greater GI symptoms correlated with reduced GMV in the occipital pole only in patients (details and correlation plots in Supplementary Figure 9). Note that multiple regression analysis performed without correction for multiple comparisons revealed an interaction effect between group and GI-symptoms in seven clusters (at *p* < 0.001, uncorrected for multiple comparisons), comprising additional frontal and temporal regions as well as the left posterior cingulate gyrus (Figure 2B, Table 3). These results are additionally visualized on axial slices (Supplementary Figure 6).

### Associations Between Gray Matter Volume and Chronic Stress

Multiple regression analysis evaluating correlations of GMV with chronic stress did not yield significant interaction effects for the analysis including patients with UC and controls (neither with FWE-correction nor with a more liberal threshold). Conversely, the same analysis in patients with IBS and controls revealed a significant interaction effect between group and chronic stress in a total of 10 clusters, encompassing the bilateral angular gyrus, bilateral temporal pole, left superior frontal gyrus (medial segment), right middle frontal gyrus, right inferior frontal gyrus (triangular part), right postcentral gyrus, right thalamus, and right inferior occipital gyrus (Table 4). Supplemental partial correlational analyses between extracted GMV for regions identified by multiple regression and chronic stress suggested that associations were consistently negative in IBS, supporting that more stress was related to lower GMV, while correlations were overall positive in healthy controls (details and correlation plots in Supplementary Figure 10). Note that multiple regression analysis performed without correction for multiple comparisons resulted in an interaction effect between group and chronic stress in 15 clusters at *p* < 0.001, comprising additional frontal regions, left middle cingulate gyrus, right anterior insula, left basal forebrain, and left caudate (Figure 2C, Table 4). These results are additionally visualized on axial slices (Supplementary Figure 7).

**Table 4:** Results of multiple regression correlating gray matter volume and chronic stress in patients with IBS and healthy controls.

**Brain region**	**H**	**k**	**TFCE**	***p*** ^*^	**x**	**y**	**z**
**IBS < HC_IBS_**							
Angular gyrus	L	2,006	2,566.1	0.001	−44	−50	48
	R	5,402	2,119.6	0.004	44	−38	41
Temporal pole	L	7,230	1,836.4	0.01	−47	8	−14
	R	6,535	2,320.7	0.002	41	8	−27
Superior frontal gyrus	L	5,205	2,010.2	0.006	−20	51	8
Middle frontal gyrus	R	428	1,565.7	0.023	36	8	54
Inferior frontal gyrus (triangular part)	R	112	1,341.4	0.045	41	32	5
Postcentral gyrus	R	957	1,481.8	0.03	44	−5	32
Thalamus proper	R	6,057	1,696.7	0.016	6	−17	−2
Inferior occipital gyrus	R	430	1,362.4	0.042	44	−75	−8
Superior frontal gyrus	R	9,925	2,010.2	<0.001	−20	51	8
Inferior frontal gyrus (orbital part)	R	158	1,323.5	0.001	35	35	0
	L	120	1,280.1	<0.001	−42	26	2
Anterior orbital gyrus	L	314	1,048.9	0.001	−30	41	−6
Precentral gyrus	R	7,445	1,565.7	<0.001	36	8	54
Middle cingulate gyrus	L	642	1,192.4	0.001	−9	−11	39
Anterior insula	R	497	1,530.2	0.001	36	−11	9
Basal forebrain	L	191	1,427.6	<0.001	−9	5	−12
Caudate	L	113	1,310	0.001	−14	20	−3

* *P-values from FWE-corrected analysis are underlined; all other values indicate results from uncorrected analysis (interaction group x chronic stress). H, hemisphere; L, left; R, right; k, cluster size; TFCE, threshold-free cluster enhancement; x, y, z, MNI coordinates; IBS, irritable bowel syndrome; HC_IBS_, matched healthy control group for IBS group*.

### Supplemental Analyses

For details on methods and results, see Supplementary Material (Chapter 2). In sum, the first set of supplemental analyses in the subsample of female UC and controls revealed no significant differences in GMV between patients and controls, but largely unchanged results of multiple regression analyses. For GI symptoms, significant interaction effects were observed in comparable clusters (Supplementary Table 1). For chronic stress, no significant interaction effects were demonstrated (neither with FWE-correction nor with a more liberal threshold), in line with the original analysis. The second set of analyses on GMV plots and results of ROI-based analyses indirectly addressing the question whether the observed GMV alterations are disease-specific are presented in the (Supplementary Figures 11, 12, respectively). Results revealed disease-specific GMV changes, especially for IBS, together with shared GMV alterations for a small subset of brain regions for both disorders.

## Discussion

Structural brain alterations in chronic pain conditions remain incompletely understood, especially in chronic visceral pain. We herein included UC as a chronic-inflammatory bowel disease and IBS as a disorder of gut-brain interactions as two distinct and clinically-relevant patient cohorts, together comprising the most prominent clinical conditions associated with chronic visceral pain and other burdening GI symptoms of the gut-brain axis. To elucidate structural brain alterations, we accomplished parallelized whole-brain voxel-based morphometry analyses in UC and IBS, each compared to an age-matched healthy control group. In addition to assessing altered GMV using analysis of covariance, multiple regression analyses were accomplished testing associations with symptom severity and chronic stress as a crucial psychological factor relevant to the pathophysiology and treatment of both conditions.

In our UC patient cohort, we observed decreased GMV in the anterior insula and middle frontal cortex when compared to age-matched healthy controls, in line with findings reported for patients with Crohn's disease (24, 25) and for mixed IBD samples including both UC and Crohn's disease patients (28, 29). Conversely, the only other existing study addressing brain morphology specifically in patients with UC found no alterations in GMV when compared to controls (30). However, while we successfully matched UC patients to controls with respect to age, our recruitment did not control for sex, resulting in an unequal distribution of males and females. A supplemental analysis testing group differences in a smaller subset of data that only included women failed to confirm group differences observed in the larger sample. This could indicate a role of sex/gender, or reflect limited statistical power due to the reduced sample size. Clearly, small sample sizes are a major limitation not only of the present study but also of existing previous work in IBD, precluding more conclusive answers on altered brain morphology in UC in remission, which may be very subtle and/or exist only in specific subsets of patients. A related concern are challenges faced by brain imaging research in IBD produced by the waxing and waning nature of symptoms and underlying inflammatory processes, and large interindividual differences in disease course and treatment, calling for decisions about inclusion and exclusion that are never unequivocal. The only other existing study specifically addressing patients with UC focused on a highly-selected sample of patients (*N* = 18) without any disease activity for at least 6 months, and with no more than one inflammatory flare since diagnosis (30). While we similarly excluded patients with active disease, the exclusion criteria for our somewhat larger sample (*N* = 31) were not as restrictive, allowing recruitment of a sample with disease ranging from full remission to low and well-managed disease activity, without restrictions with respect to number of previous flares or medication history. Consistent with this strategy, UC patients in our study reported significantly more GI symptoms, including lower abdominal pain, as well as greater psychological distress, when compared to healthy controls. This clinical presentation is arguable more representative of the typical patient population with UC outside of acute exacerbations, consistent with evidence that patients with IBD often report GI symptoms and a psychological disease impact during remission.

Bearing the critical considerations described above in mind, it is nevertheless interesting to discuss our findings suggesting possibly reduced GMV in the anterior insula and the middle frontal cortex in UC. The anterior insula is part of the salience network, which is highly relevant to pain anticipation and pain modulation in acute and chronic visceral pain [e.g., (4, 20, 23)]. Interestingly, in IBD with and without abdominal pain, resting state functional MRI revealed differences in the insula, and correlations with daily pain scores (55). Furthermore, transcranial direct current stimulation over the motor cortex demonstrably resulted in modified insula connectivity and reduced pain (56), and functional brain imaging revealed altered insula activation in anticipation of painful rectal distensions (57). The anterior insula together with frontal regions is also part of the central autonomic network, with a broad role in diverse GI sensorimotor functions along the gut-brain axis (20), including adaptive responses to the experience of recurring pain (58). This is particularly interesting given evidence supporting specific alterations in autonomic nervous system function in IBD [e.g., (59, 60)], also in relation to stress [reviewed in Labanski et al. (12)].

Our parallelized analyses in a patient cohort with IBS, which we consider an interesting disease control group for UC, revealed largely distinct and much more widespread structural brain alterations when compared to healthy controls. Brain alterations comprised both decreases as well as increases in GMV in multiple regions of the sensorimotor, central executive, and default mode networks, all demonstrably relevant to different facets of chronic visceral pain (20) and largely consistent with published findings in the literature (21, 22). We performed the present analyses with the intention to provide evidence in UC and IBS as the most prominent chronic visceral pain conditions together within one report, complementing our earlier functional brain imaging efforts in this direction (61), here aiming to discern a possible specificity of brain structural alterations to chronic visceral pain condition. While we abstained from direct patient group comparisons for methodological and conceptual reasons, the pattern of alterations in UC and IBS, respectively, when compared to controls appears to be rather dissimilar, in line with our hypothesis and further supported by supplemental ROI-based analyses. Together, these suggest mostly distinct and IBS-specific GMV alterations, with only minor putative overlap in a few subregions in UC. There exist very few neuroimaging studies that applied brain imaging techniques in IBS and IBD within one study, and ours is the first to use VBM to elucidate brain morphology. These studies collectively support disease-specific alterations (61–65), which is intriguing given the ongoing debate on overlapping and distinguishing features of these disorders (66, 67).

For a better understanding of GMV alterations, elucidating their relation with clinical as well as psychological factors is an important step. Associations of GMV changes with symptom severity have previously been demonstrated in patients with IBS and patients with Crohn's disease in terms of disease duration (24, 27, 68), pain duration (34), and daily pain scores (25). As GI symptoms are not only experienced by patients suffering from a bowel disorder, but also (obviously less frequently and/or intensively) by healthy volunteers, the question arises whether differences exist in the way these symptoms relate to GMV in patients. Results of our multiple regression analyses, specifically addressing interaction effects in patient samples and controls, support the hypothesis that the correlation between GI symptoms and brain structure is altered in patients. Differences from healthy controls were mainly observed in frontal brain regions (i.e., within the central executive network) in both patient groups. In addition, in patients with IBS, the relation of symptom severity and GMV, as expected, differed from that in healthy volunteers in regions of the sensorimotor network and default mode network. Thus, the present study confirms and expands previous findings on the relation of symptom severity and GMV in patients suffering from a chronic-inflammatory or functional bowel disorder.

In addition, the present study is the first to investigate whether structural brain measures are related to chronic stress in patients with chronic visceral pain. This question arises given the broad role of stress and stress mediators in normal visceroception (50), visceral pain sensitivity (3), visceral pain modulation (69), and altered brain processing of acute visceral pain in IBS (70). Even more importantly, stress shapes GI symptom experience and disease course both in IBS (71, 72) and IBD (37, 38), and is incorporated in treatment approaches in both conditions (73, 74). Results revealed a differential association of chronic stress with GMV in patients with IBS and healthy volunteers, encompassing numerous brain regions involved in networks relevant to the psychological modulation of visceral pain (20). In addition to regions of the sensorimotor network, central executive network, and default mode network (in which associations with symptom severity were also observed), the relation of chronic stress and GMV in regions of the salience network was significantly altered in patients with IBS, which is interesting given recent evidence indicating the unique salience of pain arising from the visceral modality (4, 5). Supplemental partial correlational analyses accomplished within each group, pointed to consistently negative associations within the group of IBS patients but not the control group, suggesting that higher chronic stress was associated with lower regional brain volumes exclusively within patients. While exploratory, these results are intriguing and may indicate that chronic stress constitutes a vulnerability factor only in patients, which in concert with additional disease-relevant mechanisms contributes to disturbed gut-brain interactions.

The same analysis of patients with UC, on the other hand, unexpectedly yielded no differences in the association of GMV changes and chronic stress. However, this negative result is difficult to interpret given the absence of group differences in chronic stress levels in our UC cohort, indicating essentially normal perceived chronic stress in this sample despite elevated clinical symptoms of anxiety as quantified with the HADS. While sample characteristics of UC were in this respect similar to an earlier study in a different sample of UC that used a comparable recruitment strategy (75), other studies from our own group (76) and other groups [e.g., (63)] reported more psychological impairment in patients, including elevated chronic stress levels. The lack of elevated chronic stress in this UC sample obviously limits the interpretation of these results, although owing to our approach to test the interaction this does not *per se* exclude an impact but rather a disease-specific differential association compared to controls. Clearly, our data do not provide conclusive answers, and hopefully inspire further study, possibly in selected patient groups presenting with higher stress levels or other impairment in psychological health, as recently accomplished by our group in a treatment trial (75), or in concert with biological measures relevant to neuroendocrine stress mediators and inflammation (76), both accomplished without concurrent brain imaging. Longitudinal studies already elucidated the relation between stress and disease course (37, 38). Including structural MRI as additional non-invasive measure in such studies appears feasible and attractive in order to further advance knowledge about the brain as “central hub” of the gut-brain axis and its interconnections with the central and peripheral stress systems, and its role in different conditions characterized by chronic visceral pain. This would promote translational efforts in the field to advance our understanding of brain measures relevant to perception and pain.

## Data Availability Statement

The datasets presented in this study can be found in online repositories. The names of the repository/repositories and accession number(s) can be found at: Neurovault (https://neurovault.org/collections/EIGBDJQK/).

## Ethics Statement

The studies involving human participants were reviewed and approved by Ethics Committee of the University Hospital Essen. The patients/participants provided their written informed consent to participate in this study.

## Author Contributions

HÖ and LK: acquired data. SE, HE, JL, and LK: designed the study. HÖ, FL, and NT: analyzed the data. HÖ and SE: wrote the first draft of the paper. SE and HE: acquired funding. All authors contributed to the interpretation of the data, revised the manuscript for critical content, and approved the final version of the manuscript.

## Funding

This work was supported by funding from the Deutsche Forschungsgemeinschaft (DFG, German Research Foundation; Project Nos. 316803389–SFB 1280 and 422744262—TRR 289). The funding organization was not involved in study design; in collection, analysis, and interpretation of data; in the writing of the report; or in the decision to submit the article for publication.

## Conflict of Interest

The authors declare that the research was conducted in the absence of any commercial or financial relationships that could be construed as a potential conflict of interest.

## Publisher's Note

All claims expressed in this article are solely those of the authors and do not necessarily represent those of their affiliated organizations, or those of the publisher, the editors and the reviewers. Any product that may be evaluated in this article, or claim that may be made by its manufacturer, is not guaranteed or endorsed by the publisher.
