# Supplementary material for: Altered Brain Structure in Chronic Visceral Pain: Specific Differences in Gray Matter Volume and Associations With Visceral Symptoms and Chronic Stress
Source: Front Neurol. 2021 Oct 20;12:733035. doi: 10.3389/fneur.2021.733035 (PMC8564184; doi:10.3389/fneur.2021.733035)
Supplement: Supplementary file 1 [file Data_Sheet_1.pdf]

Supplementary Material

Altered brain structure in chronic visceral pain:

Specific differences in gray matter volume and associations with visceral symptoms and chronic stress

Hanna Öhlmann[1], Laura Ricarda Koenen[2], Franziska Labrenz[1], Harald Engler[2], Nina Theysohn[3], Jost Langhorst[4,5], Sigrid Elsenbruch[1,6*]

[1]Department of Medical Psychology and Medical Sociology, Ruhr University Bochum, Germany

[2]Institute of Medical Psychology and Behavioral Immunobiology, Center for Translational Neuro- and Behavioral Sciences, University Hospital Essen, University of Duisburg-Essen, Germany

[3]Institute of Diagnostic and Interventional Radiology and Neuroradiology, University Hospital Essen, University of Duisburg-Essen, Germany

[4]Department for Internal and Integrative Medicine, Sozialstiftung Bamberg, Germany

[5]Department for Integrative Medicine, Medical Faculty, University of Duisburg-Essen, Germany

[6]Department of Neurology, Center for Translational Neuro- and Behavioral Sciences, University Hospital Essen, University of Duisburg-Essen, Germany

*** Correspondence:**
sigrid.elsenbruch@ruhr-uni-bochum.de

Table of contents

1 Further illustration of results from primary analyses 3

1.1 Gray matter volume alterations 3

Figure S1: Axial slices for ulcerative colitis 3

Figure S2: Gray matter volume plots for ulcerative colitis 4

Figure S3: Axial slices for irritable bowel syndrome 5

Figure S4: Gray matter volume plots for irritable bowel syndrome 6

1.2 Associations of gray matter volume with gastrointestinal symptoms and chronic stress 7

Figure S5: Axial slices for ulcerative colitis (gastrointestinal symptoms) 7

Figure S6: Axial slices for irritable bowel syndrome (gastrointestinal symptoms) 8

Figure S7: Axial slices for irritable bowel syndrome (chronic stress) 9

Figure S8: Partial correlation plots for ulcerative colitis (gastrointestinal symptoms) 10

Figure S9: Partial correlation plots for irritable bowel syndrome (gastrointestinal symptoms) 11

Figure S10: Partial correlation plots for irritable bowel syndrome (chronic stress) 12

2 Supplemental analyses 13

2.1 Controlling for sex 13

Table S1: Results of multiple regression analysis testing for differential associations of gray matter volume with gastrointestinal symptoms in female patients with ulcerative colitis and healthy controls 13

2.2 Region of interest (ROI) based analyses 14

Figure S11: Results of ROI-based analyses in patients with irritable bowel syndrome 14

Figure S12: Results of ROI-based analyses in patients with ulcerative colitis 17

1 Further illustration of results from primary analyses

1.1 Gray matter volume alterations

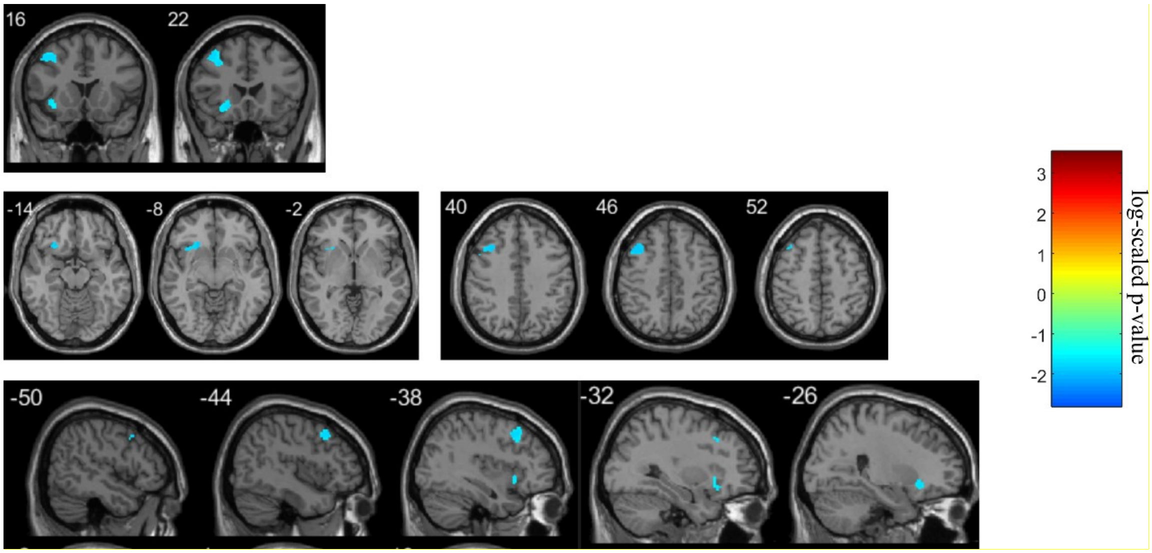

Figure S1

Axial slices of regions in which patients with ulcerative colitis had lower gray matter volume compared to healthy controls (applying FWE-correction at the significance level of p<.05; also see Figure 1A). For details, see Table 2.

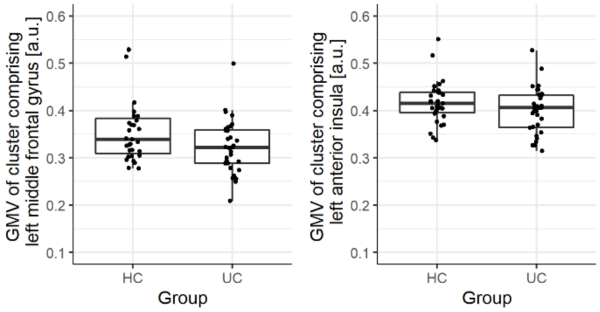

Figure S2

Extracted gray matter volume for clusters in which significantly lower gray matter volume was found in patients with ulcerative colitis compared to healthy controls (applying FWE-correction at the significance level of $p<.05$; also see Figure 1A). For details, see Table 2. Please note that these plots cannot visualize the correction for total intracranial volume, age, and sequence. GMV=gray matter volume, HC=healthy controls, UC=ulcerative colitis

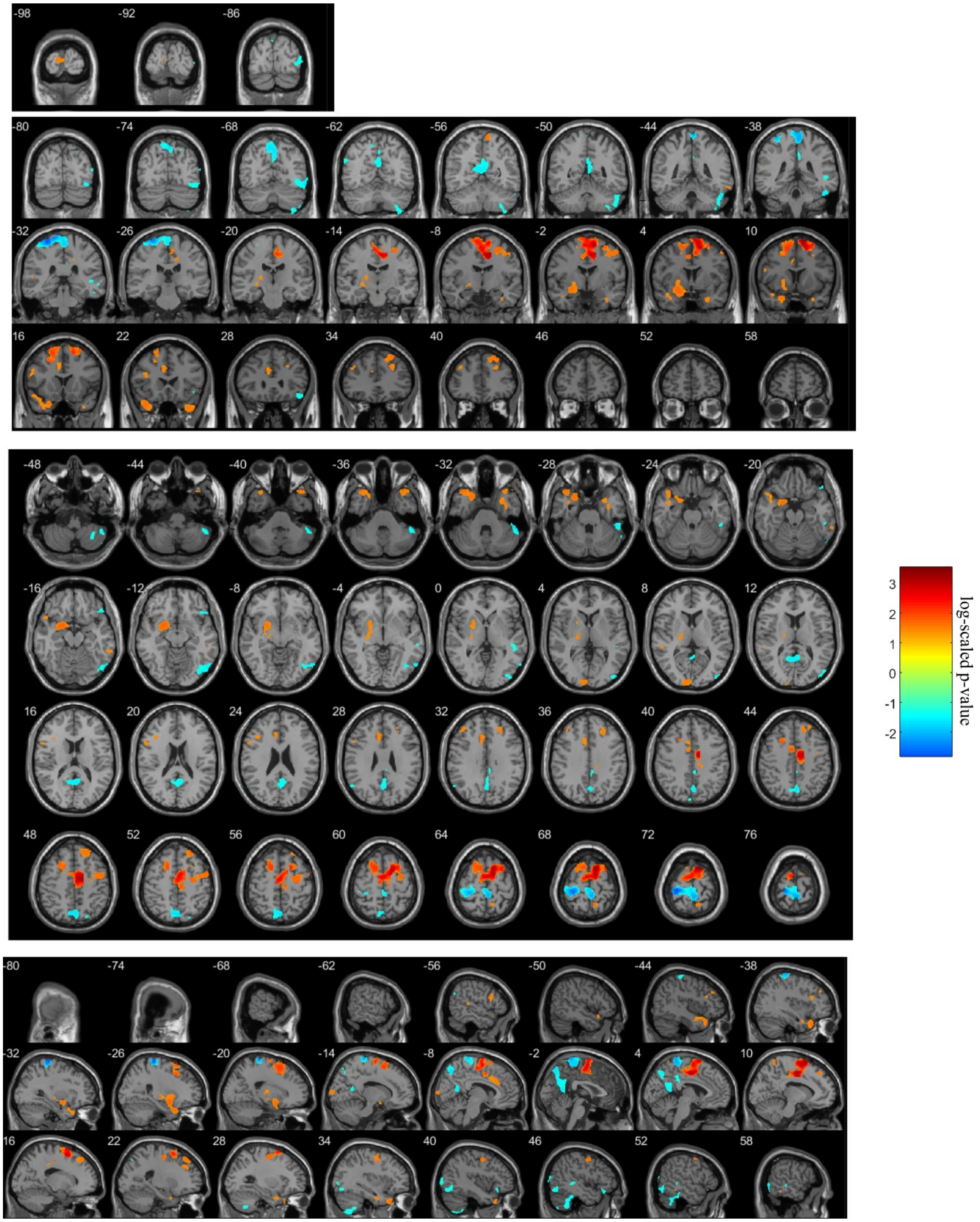

Figure S3

Axial slices of regions in which patients with irritable bowel syndrome had lower (blue color scale) or higher (red color scale) gray matter volume compared to healthy controls (applying FWE-correction at the significance level of $p<.05$; also see Figure 1B). For details, see Table 2.

(A)

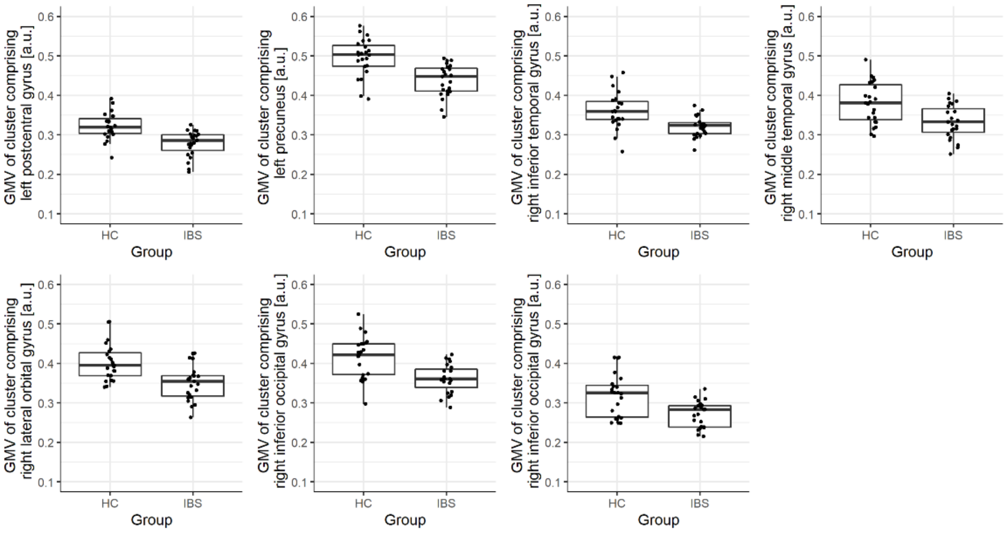

(B)

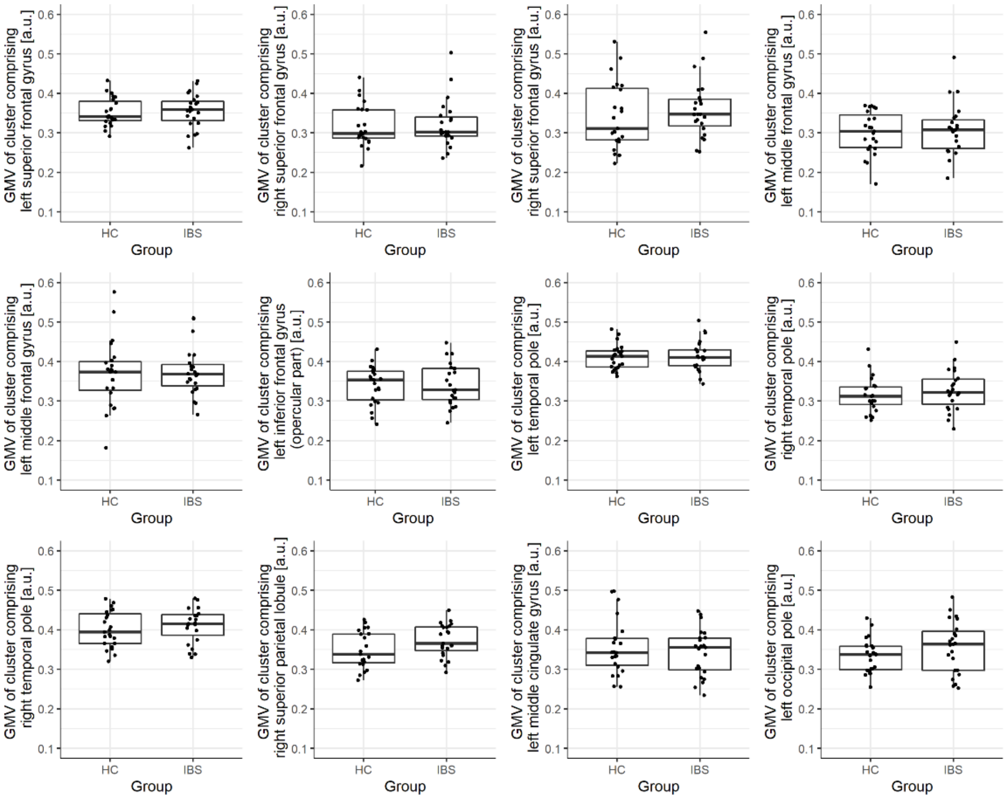

Figure S4

Extracted gray matter volume for clusters in which significantly (A) lower and (B) higher gray matter volume was found in patients with irritable bowel syndrome compared to healthy controls (applying FWE-correction at the significance level of $p<.05$; also see Figure 1B). For details, see Table 2. Please note that these plots cannot visualize the correction for total intracranial volume and age. GMV=gray matter volume, HC=healthy controls, IBS=irritable bowel syndrome

1.2 Associations of gray matter volume with gastrointestinal symptoms and chronic stress

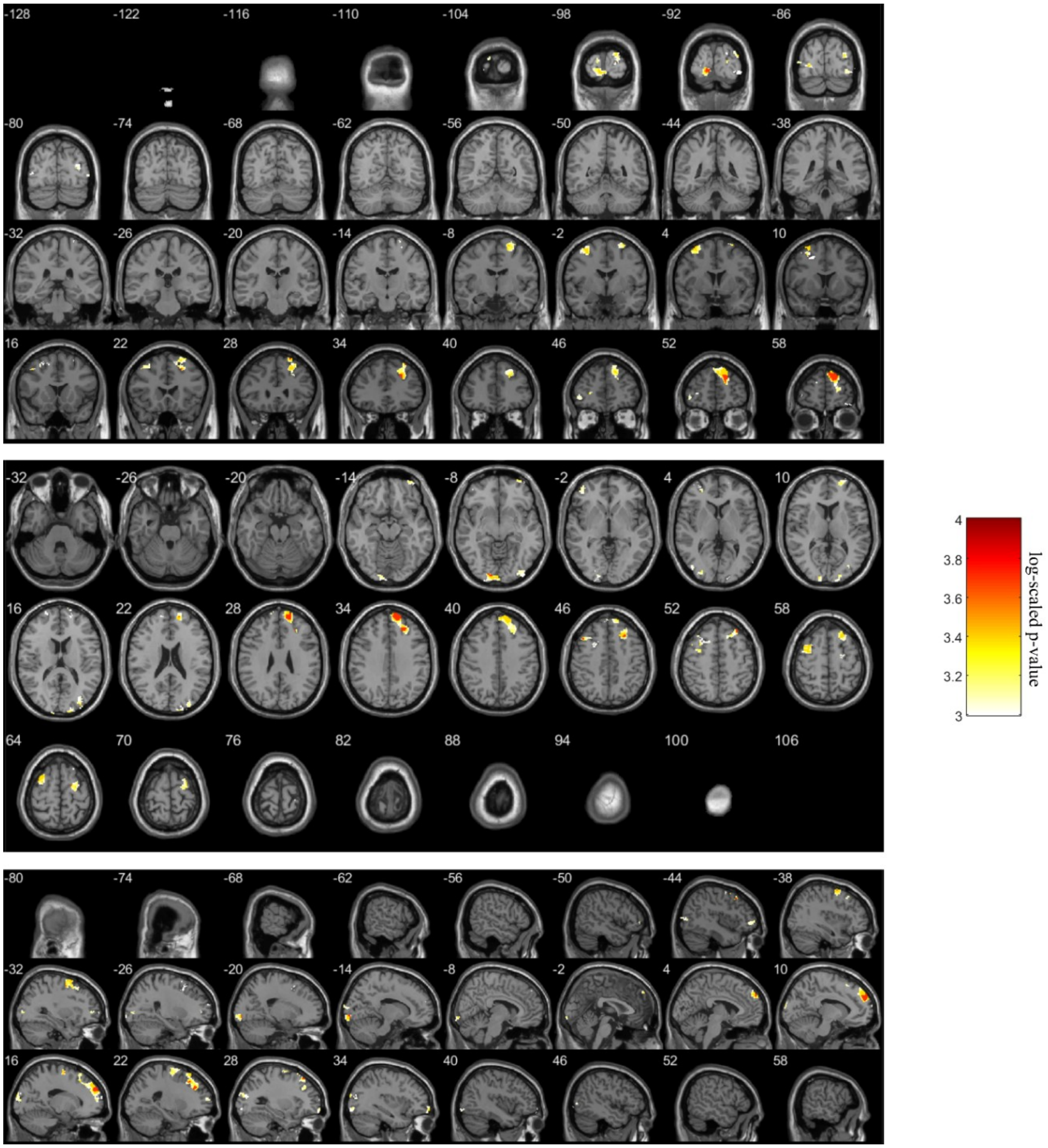

Figure S5

Axial slices of regions in which gray matter volume was differentially correlated with gastrointestinal symptoms in patients with ulcerative colitis compared to healthy controls (applying a significance level of p<.001, uncorrected for multiple comparisons; also see Figure 2A). For details, see Table 3.

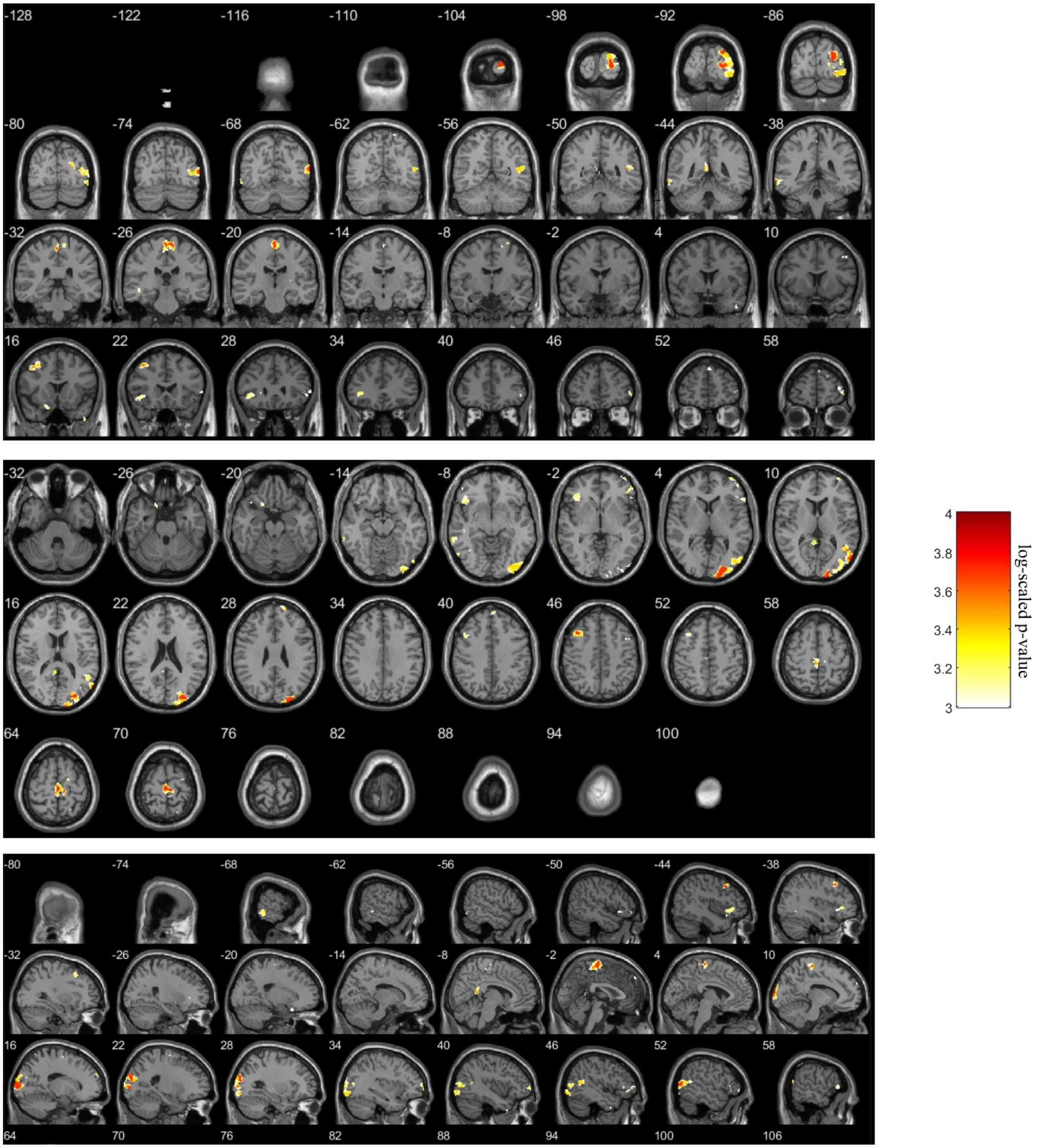

Figure S6

Axial slices of regions in which gray matter volume was differentially correlated with gastrointestinal symptoms in patients with irritable bowel syndrome compared to healthy controls (applying a significance level of $p<.001$, uncorrected for multiple comparisons; also see Figure 2B). For details, see Table 3.

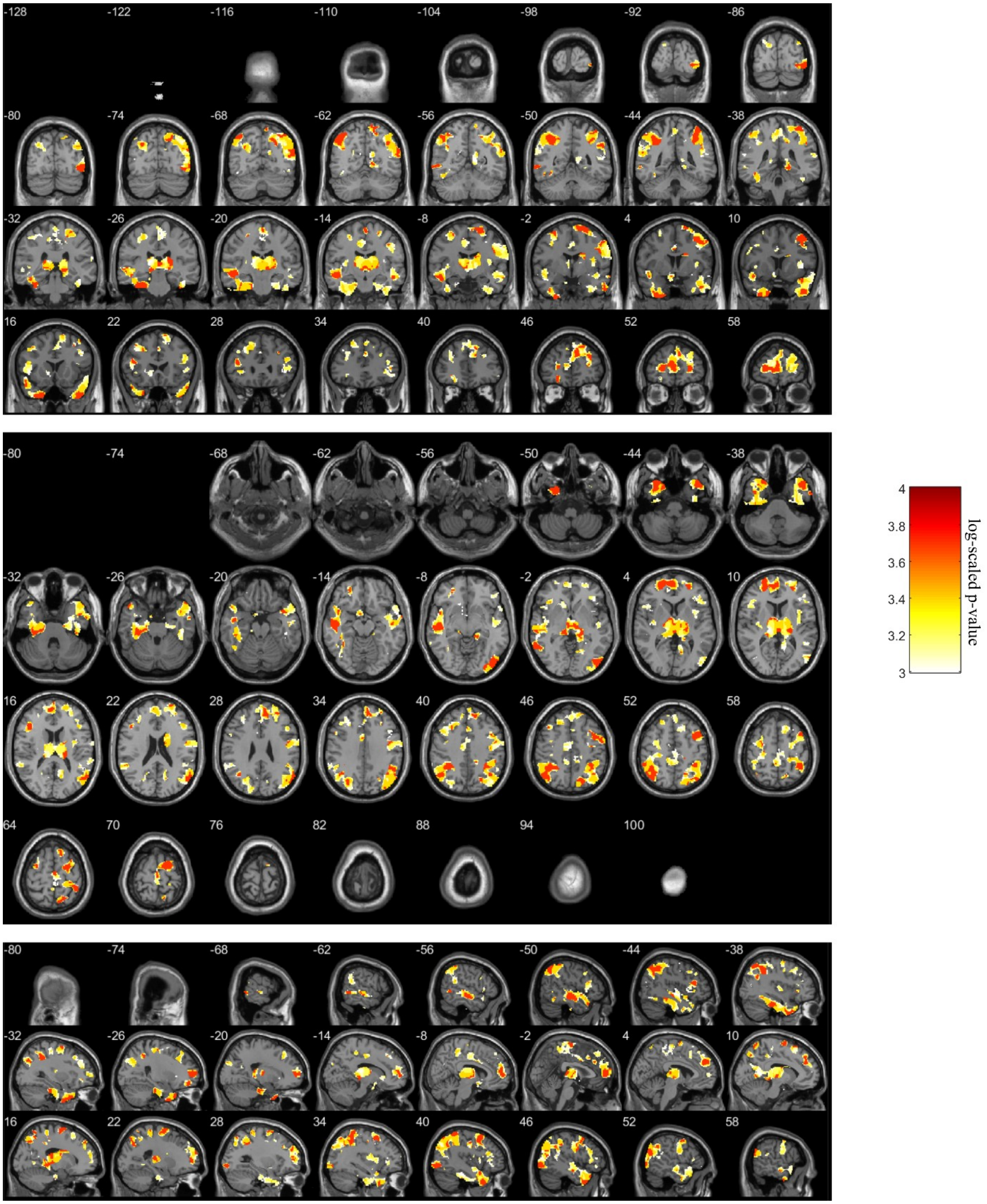

Figure S7

Axial slices of regions in which gray matter volume was differentially correlated with chronic stress in patients with irritable bowel syndrome compared to healthy controls (applying a significance level of p<.001, uncorrected for multiple comparisons; also see Figure 2C). For details, see Table 4.

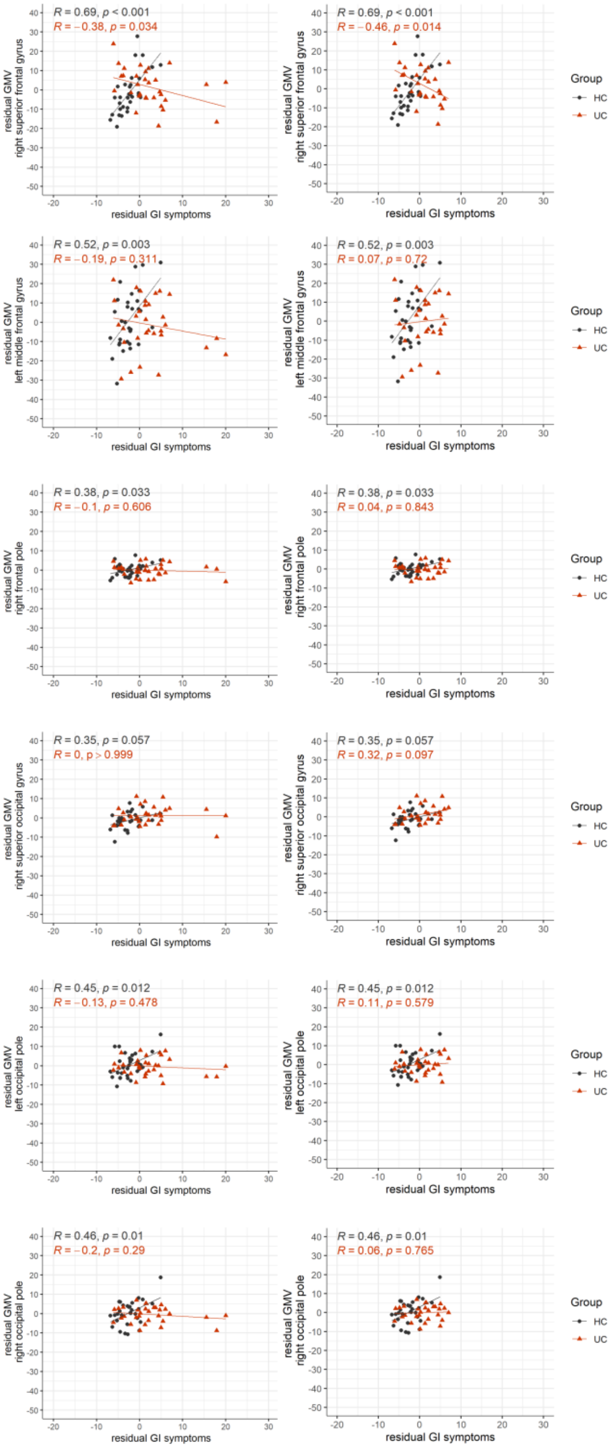

Figure S8

Results of within-group exploratory partial correlation analyses (i.e. controlling for total intracranial volume, age, and sequence) in patients with ulcerative colitis and healthy controls. The gray matter volume of the peak anatomical brain regions (Table 3) was correlated with gastrointestinal symptoms. Results are additionally provided excluding three patients with extreme values on residual GI symptoms (see plots on the right). GMV=gray matter volume, GI=gastrointestinal, HC=healthy controls, UC=ulcerative colitis

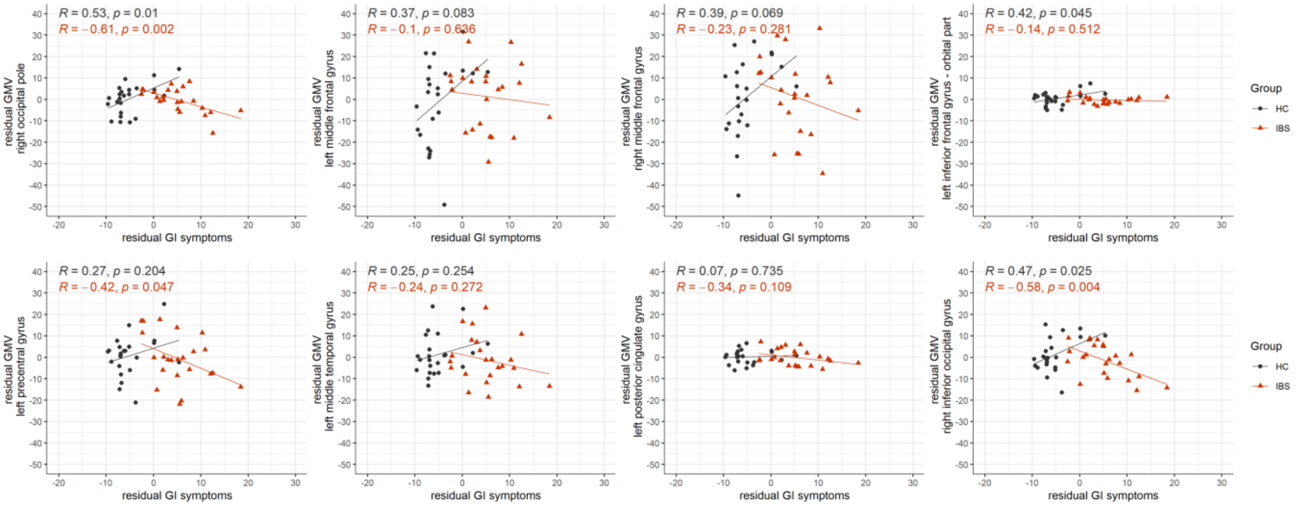

Figure S9

Results of within-group exploratory partial correlation analyses (i.e. controlling for total intracranial volume and age) in patients with irritable bowel syndrome and healthy controls. The gray matter volume of the peak anatomical brain regions (Table 3) was correlated with gastrointestinal symptoms. GMV=gray matter volume, GI=gastrointestinal, HC=healthy controls, IBS=irritable bowel syndrome

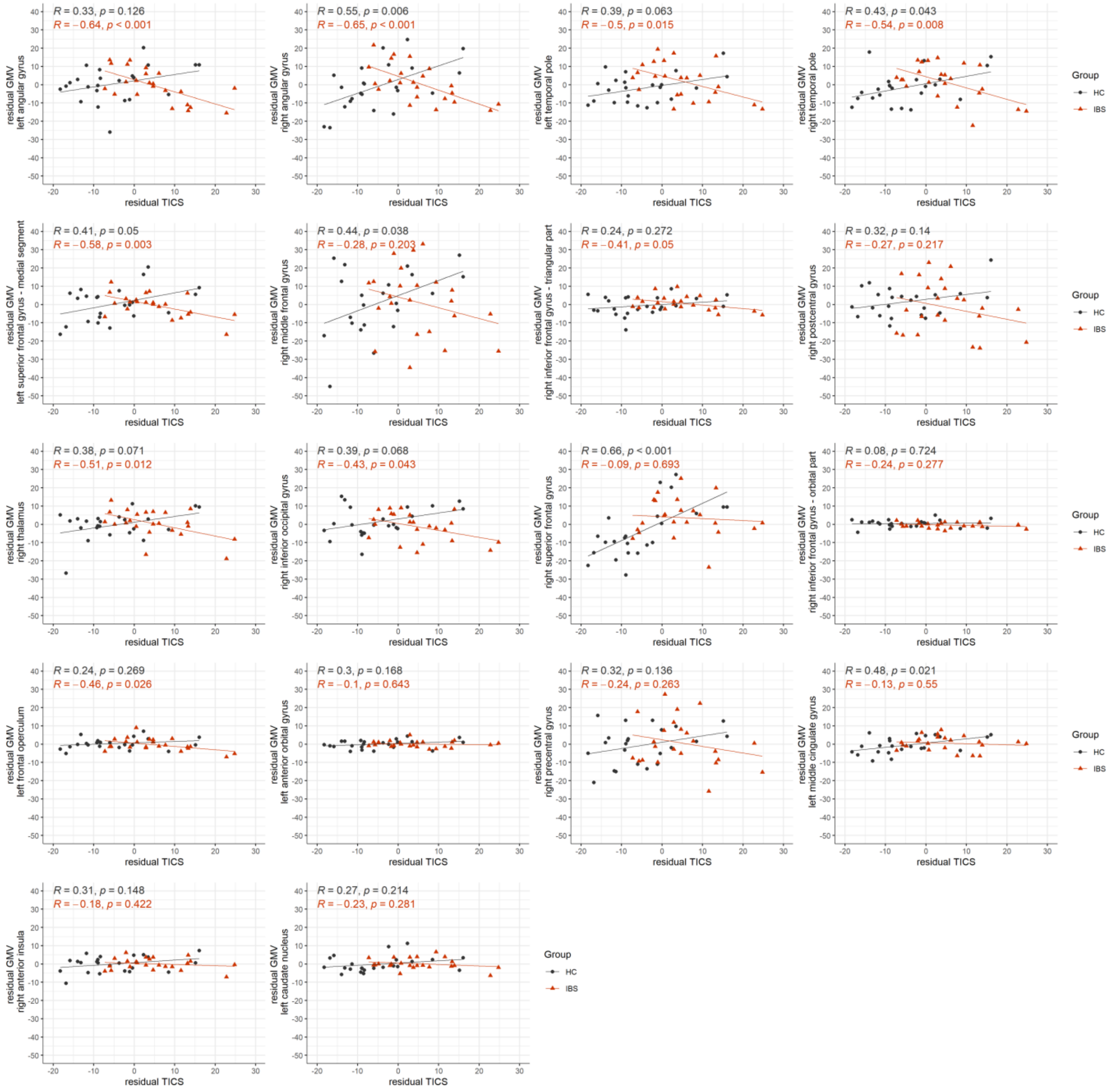

Figure S10

Results of within-group exploratory partial correlation analyses (i.e. controlling for total intracranial volume and age) in patients with irritable bowel syndrome and healthy controls. The gray matter volume of the peak anatomical brain regions (Table 4) was correlated with chronic stress. GMV=gray matter volume, GI=gastrointestinal, HC=healthy controls, IBS=irritable bowel syndrome

2 Supplemental analyses

2.1 Controlling for sex

Table S1

Results of whole-brain multiple regression correlating gray matter volume with gastrointestinal symptoms in female patients with UC (*N*=26) compared to healthy controls (*N*=26)

Brain region	H	k	TFCE	p*	x	y	z
**UC < HC$_{UC}$**							
Superior frontal gyrus	R	832	1695.90	.013	18	41	36
	L	381	793.50	<.001	-11	60	26
Middle frontal gyrus	L	555	985.97	<.001	-32	60	3
	L	554	898.37	<.001	-33	2	57
Occipital pole	R	1241	1287.53	.001	30	-87	18
Calcarine cortex	L	159	941.48	.001	-18	-95	-5
Inferior temporal gyrus	R	177	772.52	.001	51	-45	-14

*P-values from FWE-corrected analyses are underlined; all other values indicate results from uncorrected analyses (interaction group x GI symptoms). H = hemisphere, L = left, R = right, k = cluster size, TFCE = threshold-free cluster enhancement, x, y, z = MNI coordinates, UC = ulcerative colitis, HC$_{UC}$ = matched healthy control group for UC group

2.2 Region of interest (ROI) based analyses

While we abstained from direct patient group comparisons for methodological and conceptual reasons, we aimed to indirectly address whether patterns of gray matter volume (GMV) alterations in patients with ulcerative colitis (UC) and irritable bowel syndrome (IBS) are disease-specific following a valuable suggestion. To achieve this, for each patient group and matched healthy controls, GMV was extracted and plotted for every cluster in which GMV alterations were found in the other patient group compared to matched healthy controls (see Figure S11 for UC-clusters examined in IBS patients, and Figure S12 for IBS-clusters examined in UC patients). For details on the underlying clusters, see Table 2. In addition, region of interest (ROI) based ANCOVAs were run in patients with UC and IBS, respectively, and matched healthy controls. Again, the total intracranial volume, age, and sequence (where appropriate) were entered as covariates of no interest. Masks were created for the clusters in which GMV alterations were observed in the other patient group and used as ROI in separate analyses to test whether similar GMV alterations might be present in the patient group under examination, which did not show up in whole-brain analyses for statistical reasons. Results are provided for patients with irritable bowel syndrome (Figure S11) and ulcerative colitis (Figure S12).

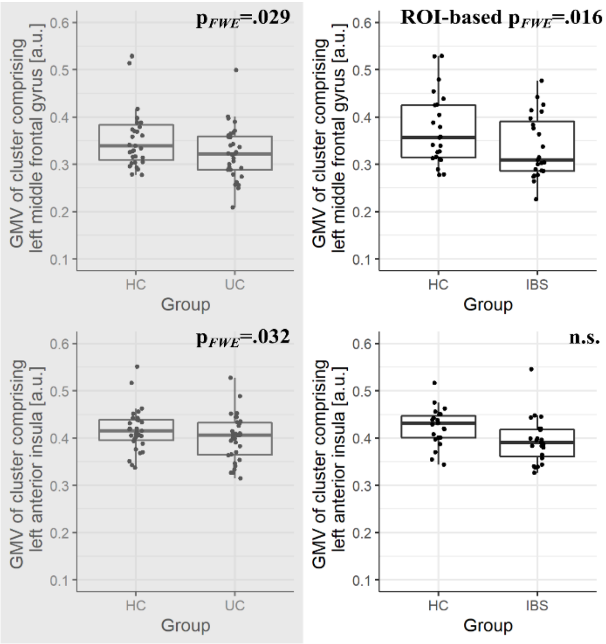

Figure S11

For patients with irritable bowel syndrome and matched healthy controls, gray matter volume was extracted and plotted for every cluster in which lower gray matter volume was found in patients with ulcerative colitis compared to matched healthy controls in whole-brain analysis (details on clusters in Table 2). Results of ROI-based analyses are provided in the upper right corner (n.s.=non-significant). For the sake of completeness, the original gray matter volume plots (with original p-values) for patients with ulcerative colitis are shown as well (highlighted in gray; same plots as in Figure S2). GMV=gray matter volume, HC=healthy controls, UC=ulcerative colitis, IBS=irritable bowel syndrome, FWE=family-wise error

(A)

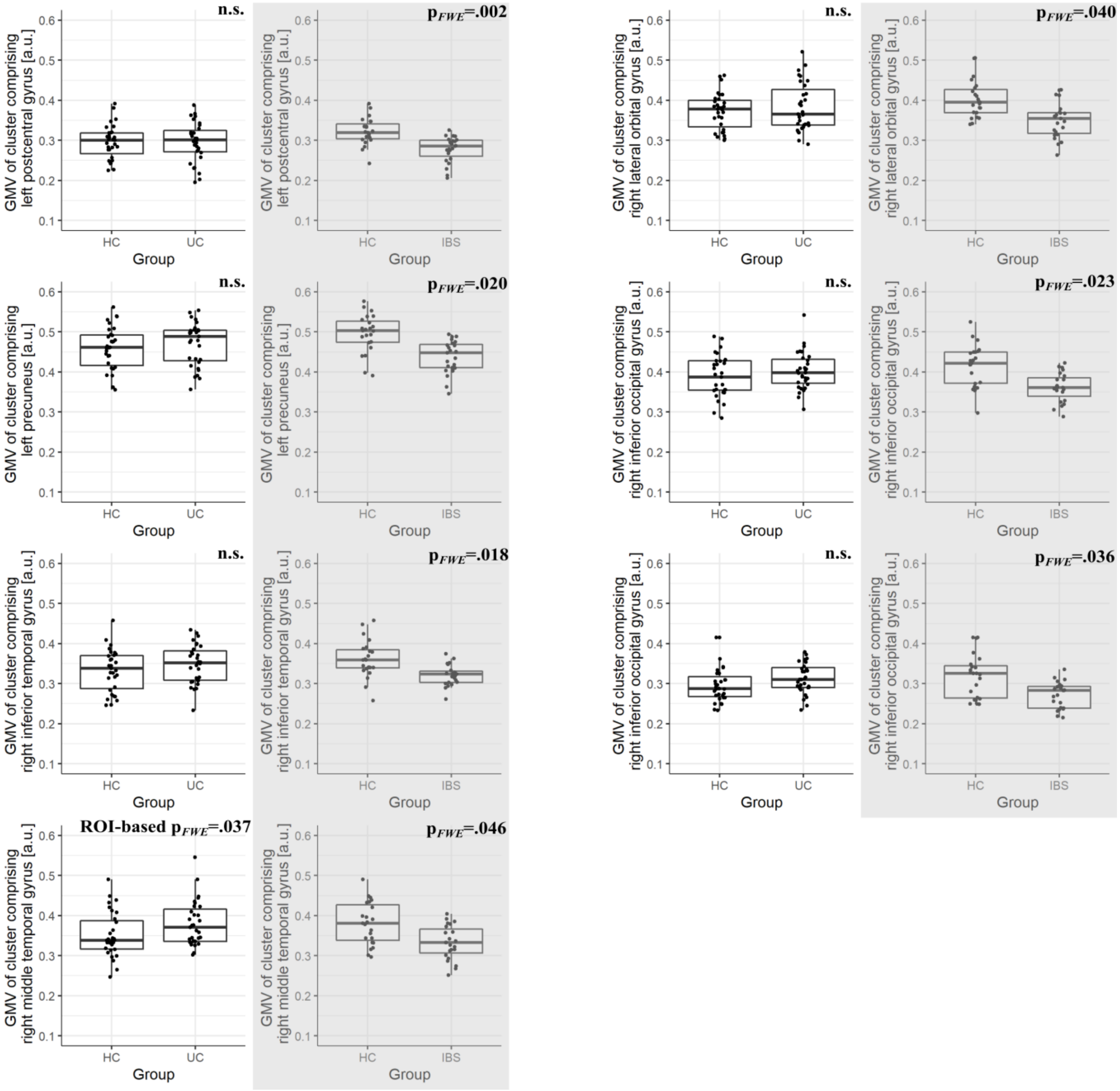
n.s.
pFWE=.002
pFWE=.040
pFWE=.020
pFWE=.023
pFWE=.018
pFWE=.036
ROI-based pFWE=.037
pFWE=.046
GMV of cluster comprising left postcentral gyrus [a.u.]
GMV of cluster comprising right lateral orbital gyrus [a.u.]
GMV of cluster comprising left precuneus [a.u.]
GMV of cluster comprising right inferior occipital gyrus [a.u.]
GMV of cluster comprising right inferior temporal gyrus [a.u.]
GMV of cluster comprising right middle temporal gyrus [a.u.]
Group
HC
UC
IBS
0.1
0.2
0.3
0.4
0.5
0.6

(B)

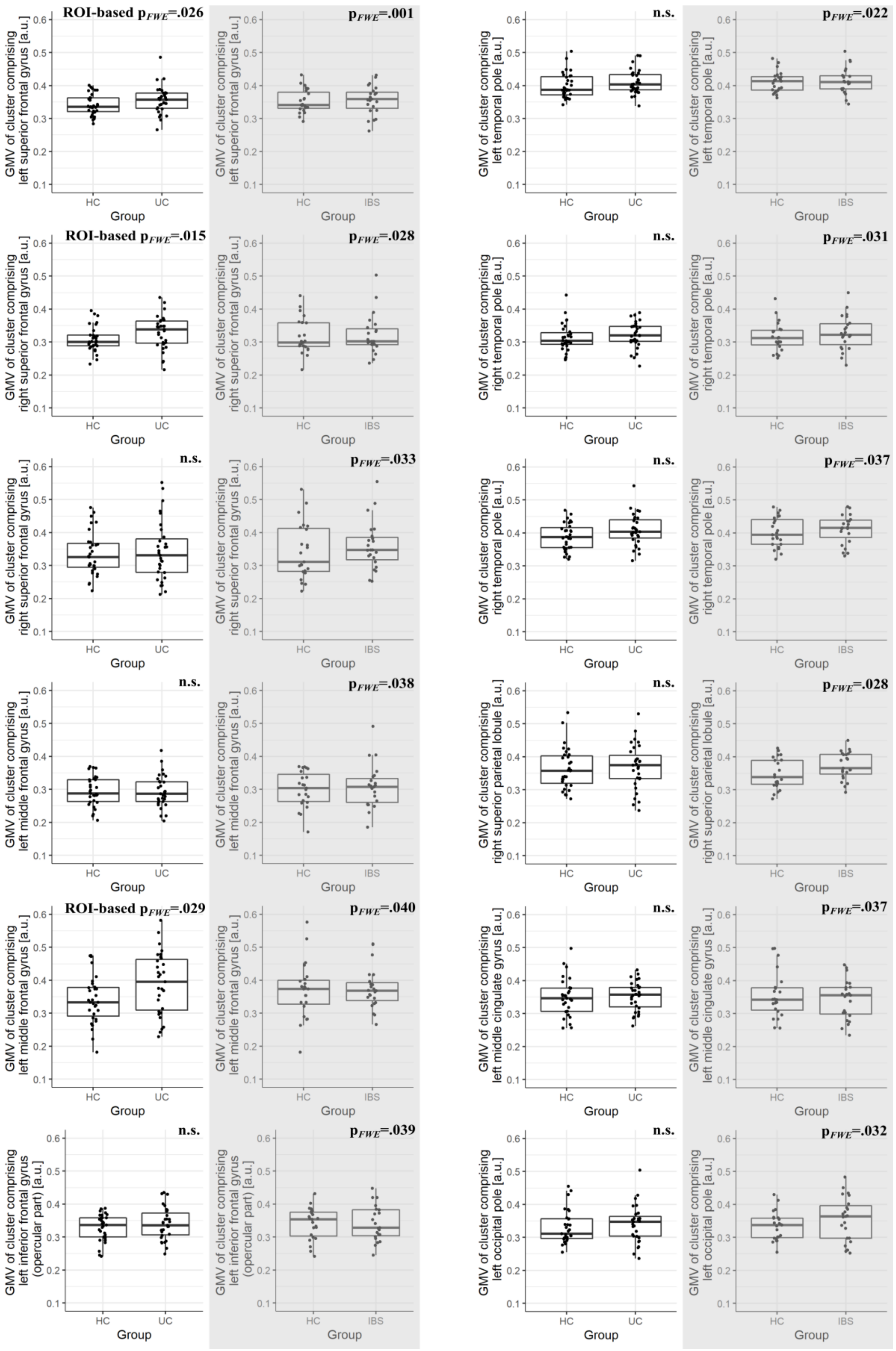
ROI-based p_{FWE}=.026
p_{FWE}=.001
n.s.
p_{FWE}=.022
GMV of cluster comprising left superior frontal gyrus [a.u.]
GMV of cluster comprising left temporal pole [a.u.]
ROI-based p_{FWE}=.015
p_{FWE}=.028
n.s.
p_{FWE}=.031
GMV of cluster comprising right superior frontal gyrus [a.u.]
GMV of cluster comprising right temporal pole [a.u.]
n.s.
p_{FWE}=.033
n.s.
p_{FWE}=.037
GMV of cluster comprising right superior frontal gyrus [a.u.]
GMV of cluster comprising right temporal pole [a.u.]
n.s.
p_{FWE}=.038
n.s.
p_{FWE}=.028
GMV of cluster comprising left middle frontal gyrus [a.u.]
GMV of cluster comprising right superior parietal lobule [a.u.]
ROI-based p_{FWE}=.029
p_{FWE}=.040
n.s.
p_{FWE}=.037
GMV of cluster comprising left middle frontal gyrus [a.u.]
GMV of cluster comprising left middle cingulate gyrus [a.u.]
n.s.
p_{FWE}=.039
n.s.
p_{FWE}=.032
GMV of cluster comprising left inferior frontal gyrus (opercular part) [a.u.]
GMV of cluster comprising left occipital pole [a.u.]
0.1
0.2
0.3
0.4
0.5
0.6
HC
UC
IBS
Group

Figure S12

For patients with ulcerative colitis and matched healthy controls, gray matter volume was extracted and plotted for every cluster in which (A) lower or (B) higher gray matter volume was found in patients with irritable bowel syndrome compared to matched healthy controls in whole-brain analysis (details on clusters in Table 2). Results of ROI-based analyses are provided in the upper right corner (n.s.=non-significant). For the sake of completeness, the original gray matter volume plots (with original p-values) for patients with irritable bowel syndrome are shown as well (highlighted in gray; same plots as in Figure S4). GMV=gray matter volume, HC=healthy controls, UC=ulcerative colitis, IBS=irritable bowel syndrome, FWE=family-wise error
